# Synapse-specific catecholaminergic modulation of neuronal glutamate release

**DOI:** 10.1073/pnas.2420496121

**Published:** 2024-12-30

**Authors:** Dariya Bakshinska, William YuChen Liu, Ryan Schultz, R. Steven Stowers, Adam Hoagland, Caroline Cypranowska, Cherise Stanley, Susan H. Younger, Zachary L. Newman, Ehud Y. Isacoff

**Affiliations:** ^a^Helen Wills Neuroscience Institute, University of California Berkeley, Berkeley, CA 94720; ^b^Department of Neuroscience and Molecular & Cell Biology, University of California, Berkeley, CA 94720; ^c^Department of Microbiology & Cell Biology, Montana State University, Bozeman, MT 59717; ^d^Weill Neurohub, University of California Berkeley, Berkeley, CA 94720; ^e^Molecular Biophysics and Integrated BioImaging Division, Lawrence Berkeley National Laboratory, Berkeley, CA 94720

**Keywords:** synapse, QuaSOR, octopamine, Unc13, glutamate

## Abstract

Norepinephrine in vertebrates and its invertebrate analog, octopamine, regulate the activity of neural circuits. We find that, when hungry, *Drosophila* larvae switch activity in type II octopaminergic motor neurons (MNs) to high-frequency bursts, which coincide with locomotion-driving bursts in type I glutamatergic MNs that converge on the same muscles. Optical quantal analysis across hundreds of synapses simultaneously reveals that octopamine potentiates glutamate release by tonic type Ib MNs, but not phasic type Is MNs, and occurs via the G_q_-coupled octopamine receptor (OAMB). OAMB is more abundant in type Ib terminals and acts through diacylglycerol and its target Unc13A, a key component of the glutamate release machinery. Potentiation varies significantly—by up to 1,000%—across synapses of a single Ib axon, with synaptic Unc13A levels determining both release probability and potentiation. We propose that a dual molecular mechanism—an upstream neuromodulator receptor and a downstream transmitter release controller—fine-tunes catecholaminergic modulation so that strong tonic synapses exhibit large potentiation, while weaker tonic and all phasic synapses maintain consistency, yielding a sophisticated regulation of locomotor behavior.

## Significance

We identify a two-component presynaptic molecular mechanism involving an upstream octopamine receptor and a downstream promoter of transmitter release (Unc13A), that determines whether a synapse is subject to modulation. Strong, Unc13A-rich synapses in tonic type Ib axons are potentiated by octopamine released from type II motor neurons, while weak, Unc13A-poor Ib synapses are not. Additionally, there is no modulation of synapses in phasic type Is axons. This modulatory specialization, which increases release by as much as 10-fold at some synapses, results in substantial overall potentiation for the type Ib synapse population but does not affect the majority of glutamatergic synapses, leaving the system open to multiple forms of regulation of locomotory behavior.

Catecholamines, such as norepinephrine and dopamine, are critical neuromodulators that control neural circuit function, brain state, and behavior (1, 2). In vertebrates, these neuromodulators exert a widespread influence due to the extensive projection patterns of norepinephrine axons (from *locus coeruleus*) and dopamine axons (from basal ganglia) to many targets within the brain (1). In insects, a similarly broad projection pattern is observed for dopamine axons (3) and for axons that release the invertebrate norepinephrine analog octopamine (OA) (4).

Analogous to the functions of norepinephrine in the vertebrate brain, OA in *Drosophila* regulates locomotion (5), courtship behavior (6), sleep (7), olfactory (8), and aversive (9) learning, as well as many other behaviors (10). For each of these actions, the modulation can occur at multiple sites in the relevant neural circuit in both larval and adult developmental stages. In the locomotion of *Drosophila* larva, this includes sites in the brain and the ventral nerve cord as well as directly at the neuromuscular junction (NMJ) of the body wall muscles that mediate movement. The circuit and cellular mechanisms of OA modulation in the central nervous system are difficult to study. We therefore turned to the excitatory synapse of the *Drosophila* larval NMJ. Each muscle receives input from two glutamatergic type I motor neurons (MNs): low release probability “tonic” type Ib and high release probability “phasic” type Is MNs (11, 12). Type I MNs synapse onto muscles near two other MN types: type II MNs, which release glutamate and OA, and type III MNs, which release neuromodulatory peptides onto subsets of muscle groups (11). Whereas small molecule transmitters such as glutamate and OA are released from small clear vesicles at all frequencies of action potential firing, peptides are released from dense core vesicles, only at high firing frequencies (13, 14). The neuromodulatory action of all of these transmitters increases with firing frequency due to the kinetics of activation and deactivation of their G protein-coupled receptors (GPCRs) and the rise and fall of components in their signaling cascade (15).

Larval starvation over hours increases locomotion in a manner that depends on intact type II MNs, and optogenetic stimulation of type II MNs, also over hours, increases locomotion (16). Consistent with this, OA increases the growth of type I MN axons, indicating that prolonged elevation of type II MN activity increases the number of excitatory glutamatergic synapses and thus locomotory drive (16–18). OA also has the acute effect of increasing type I MN evoked excitatory postsynaptic potential (EPSP) amplitude over minutes (16), but the site of this modulation (presynaptic versus postsynaptic) and the underlying signaling mechanism are not known. Moreover, the in vivo firing dynamics of type II MNs have not been measured, leaving it unclear whether its timing is compatible with fast octopaminergic modulation of glutamatergic transmission.

Combining GCaMP imaging of type II MN activity with SynapGCaMP imaging of glutamatergic type I MN synaptic transmission in *Drosophila* larvae during restrained locomotion, we find that type II MNs increase activity in hungry animals and burst during bouts of type I MN synaptic transmission, a timing compatible with OA modulation of glutamatergic transmission. Optogenetic inhibition of type II MNs rapidly reduces locomotion, consistent with a model of rapid octopaminergic potentiation of glutamatergic neuromuscular drive.

Given the differences between the two type I MNs and the wide diversity in transmission properties between synapses of each MN type (12, 19, 20), we set out to ask whether OA does indeed modulate transmission, and, if so, to determine whether modulation is uniform across all type I MN synapses and to define its molecular mechanism.

Quantal superresolution optical imaging at hundreds of identified type I MN synapses (19, 20) revealed that acute application of OA potentiates the probability of glutamate release within minutes. RNAseq results show that type I MNs express several OA activated GPCRs. RNAi knockdown of one of these, the G_q_-coupled OA receptor OAMB, eliminates the OA-induced boost of release. Consistent with this, OA modulation depends on phospholipase C (PLC) and on uncoordinated 13A (Unc13A), a key component of the transmitter release machinery, which is a known target of modulation by the PLC enzymatic product diacylglycerol (DAG). Presynaptic potentiation by OA is mimicked by the membrane-permeable DAG analog PdBU and is eliminated by either RNAi knockdown of Unc13A or replacement of wildtype Unc13A with a mutant Unc13A that cannot bind to DAG. The modulatory effect of OA varies greatly between type Ib synapses of the same axon, and the degree of potentiation is proportional to the level of Unc13A at the synapse. Strikingly, modulation by OA occurs only at the synapses of type Ib MNs and not in those of type Is phasic MNs that innervate the same muscles.

Thus, a two-component molecular mechanism, comprising the upstream OAMB receptor and the downstream Unc13A promoter of transmitter release, determines whether a synapse is subject to modulation. Only strong Unc13A-rich synapses in tonic type Ib axons undergo potentiation while weak Unc13A-poor Ib synapses do not. Additionally, synapses in phasic type Is axons show no modulation. This modulatory specialization, capable of enhancing release by as much as 10-fold, leads to substantial overall potentiation of the type Ib synapse population. However, it leaves the majority of glutamatergic synapses untouched, preserving their capacity to respond to other signals, thereby maintaining the system’s flexibility for diverse locomotor control.

## Results

### Neural Activity in Octopaminergic Type II and Glutamatergic Type I MNs During Locomotion.

**Fig. 1.**
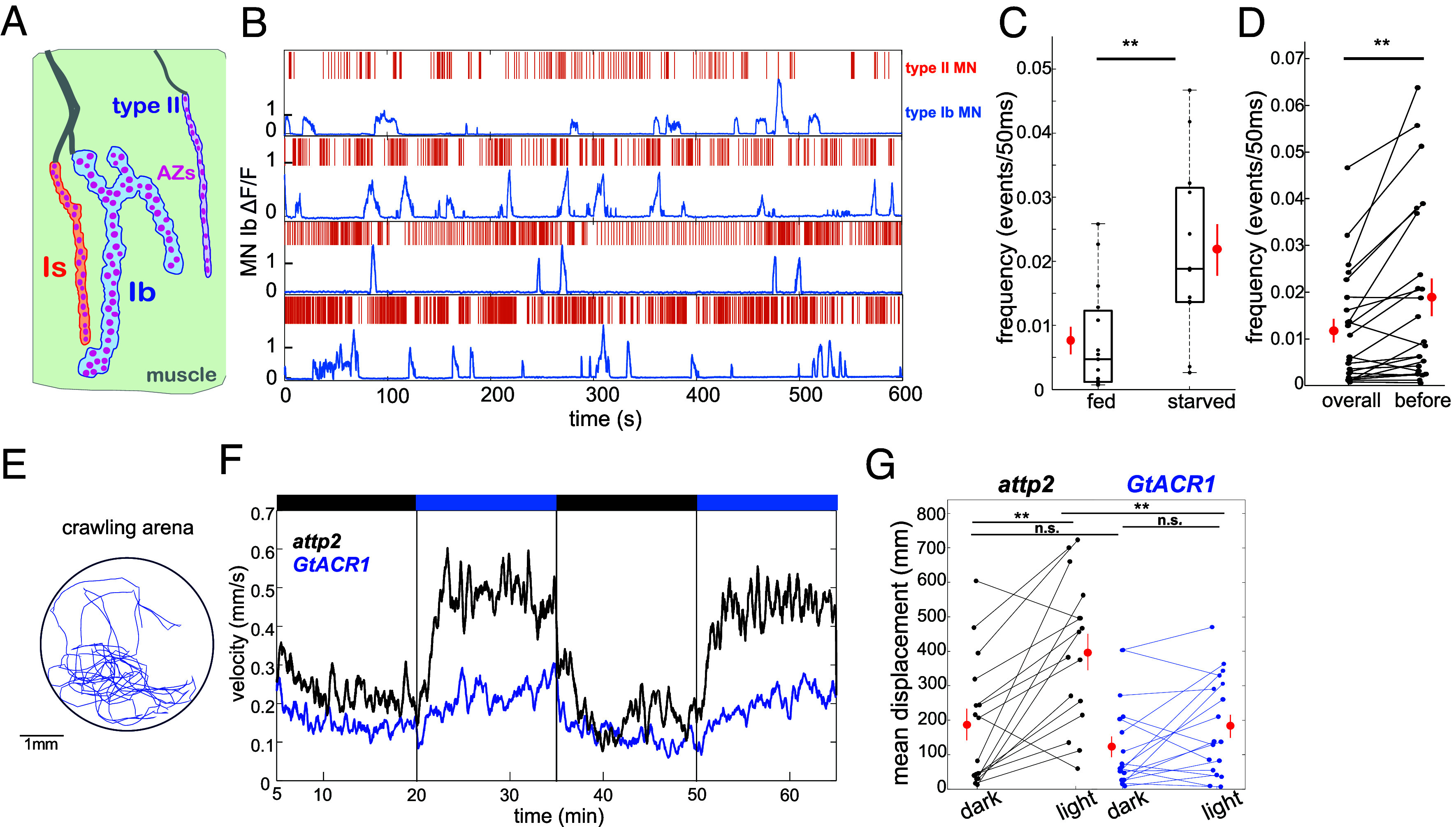
Type II MN firing frequency increases before Ib bursts and inhibition of type II MNs reduces locomotion. (*A*) Schematic depicting the *Drosophila* NMJ illustrating type II MN (purple), type I MNs: Ib (blue), and Is (orange) synapsing onto the same muscle. Individual active zones (AZs) are shown in magenta. (*B*) Examples from 4 starved animals showing type II MN activity, measured by cytosolic GCaMP6f (*Upper* orange ticks; each tick represents the first frame of a type II MN firing bout) and corresponding Ib MN synaptic transmission bout, measured by SynapGCaMP6f concentrated in the muscle postsynaptic density (lower blue trace). (*C*) Box plot showing frequency (# of events/50 ms) of type II firing in fed (n = 15) vs. starved animals (n = 12). Red points are mean ± S.E.M. (**P <0.01 by the Wilcoxon signed-rank test.) (*D*) Frequency of type II MN firing in 8 s prior to a Ib MN bout (before) compared to firing over the entire 600 s recording (overall) (n = 23). Red points are mean ± S.E.M. (**P <0.01 by the Wilcoxon signed-rank test.) (*E*) Example a larval track registered in the locomotion assay. (*F*) Mean crawling velocity of control *attp2* larvae (black trace, n = 16) and larvae expressing the inhibitory *GtACR1* in type II MNs (blue trace, n = 18). Recording starts at 5 min. to account for an adjustment period. Black bars above represent dark; blue bars represent illumination with 505 nm light. (*G*) Mean displacement for each animal in (*F*), across the two dark and two light (505 nm) periods for *attp2* (n = 16) and *GtACR1* (n = 18) animals. Red points are mean ± S.E.M. (**P <0.01; n.s., not significant by Student’s paired *t*-test).

In the *Drosophila*, muscle cells receive input from two glutamatergic type I MNs: Ib (big) and Is (small) (21), both of which form strings of boutons, each containing multiple synapses (Fig. 1*A*). Converging nearby on the same muscles there is also a type II MN that releases OA (22). Given that GPCR signaling tends to operate over hundreds of milliseconds to seconds (15), one would expect that the activity of type II MNs would need to immediately precede or coincide with that of type I MNs to modulate type I MN synaptic transmission. To assess this, we simultaneously measured the activity of type Ib MN synaptic transmission and type II MN activity during locomotion (Fig. 1*B* and *SI Appendix*, Fig. S1*C*). We observed native activity in a linear PDMS chamber that restricts the larva to crawl in place, and subsequently used movement correction to follow each NMJ over time (*SI Appendix*, Fig. S1 *A–C*) (12). The timing of type Ib MN activity was determined by measuring glutamatergic transmission postsynaptically with SynapGCaMP6f, under the control of the myosin heavy chain (MHC) promoter (MHC-SynapGCaMP6f), which localizes to the postsynaptic density and detects Ca^2+^ influx through postsynaptic (muscle) ionotropic glutamate receptors (12, 23, 24). The activity of type II MNs was measured in their terminal axons using cytoplasmic GCaMP6f (*SI Appendix*, Fig. S1*B*). The type Ib, Is, and II axons are positioned near each other on the muscle, making it plausible that OA released by the type II MNs could reach the type I MN synapses (Fig. 1*A* and *SI Appendix*, Figs. S1*B* and S6*B*). Despite their proximity, the axon terminals are easily distinguishable morphologically (*SI Appendix*, Figs. S1*B* and S6*B*).

As shown earlier (12), type Ib MNs alternated between quiescence and bursts of activity that drive contraction (Fig. 1B and *SI Appendix*, Fig. S1*C*). Type II MN activity ranged between low frequency (~0.02 to 0.5 Hz) tonic firing and burst firing (with intraburst frequency of up to 20 Hz) (Fig. 1*C* and Movie S1). Additionally, type II MN firing was increased in starved animals (Fig. 1*C*) and was elevated just before and/or during type Ib MN transmission bouts (Fig. 1 *B* and *D*). This timing is compatible with OA modulation of glutamatergic transmission.

Extended optogenetic stimulation of type II MNs or prolonged starvation increases locomotion (16, 25, 26). Furthermore, this effect of starvation is eliminated by type II MN cell death and is linked with the growth of type I MN axons. This suggests that elevated type II MN activity slowly increases the number of type I MN synapses and thereby boosts locomotion (16). We then asked whether the activity of type II MNs, of the kind we observed above, boosts locomotion over a short time scale by modulating transmission at existing synapses. To test this, we turned to optogenetic inhibition of type II MNs during a locomotion assay (Fig. 1 *E*–*G* and *SI Appendix*, Fig. S1 *E* and *F*). We expressed the anion-selective channelrhodopsin, GtACR1 (27), exclusively in type II MNs, using a split-Gal4 driver (28) (w^1118^; R31C03-p65.AD/Tdc2-Gal4.DBD; 20x-UAS-GtACR1.d.EYFP/+) (*SI Appendix*, Fig. S1*D*). Control larvae responded to illumination with blue light (505 nm) by increasing locomotion speed (Fig. 1*F*, black trace and Fig. 1 *G*, *Left* panel), consistent with prior observations that light promotes locomotion (29). Larvae expressing GtACR1 showed a significantly smaller increase in locomotion (Fig. 1*F*, blue trace and Fig. 1 *G*, *Right* panel). This locomotory suppression was evident within 1 min of the start of illumination, as early as one can detect a change in locomotor trajectory (Fig. 1*F*). Together, these observations suggest that hunger (or blue light) increases type II MN firing, which, in turn, rapidly increases type I MN glutamatergic drive for locomotion.

### OA Differentially Potentiates Release at Ib Synapses and Not at Is Synapses.

Our observations that type II MN activity comodulates with type Ib MN activity and that inhibition of type II MNs reduced locomotion are consistent with the known acute potentiation by OA of excitatory junction potential amplitude (16). Although it is known that OA receptors are found on both sides of the synapses, the site and mechanism of the acute potentiation by OA that we observe is not known (5, 16).

**Fig. 2.**
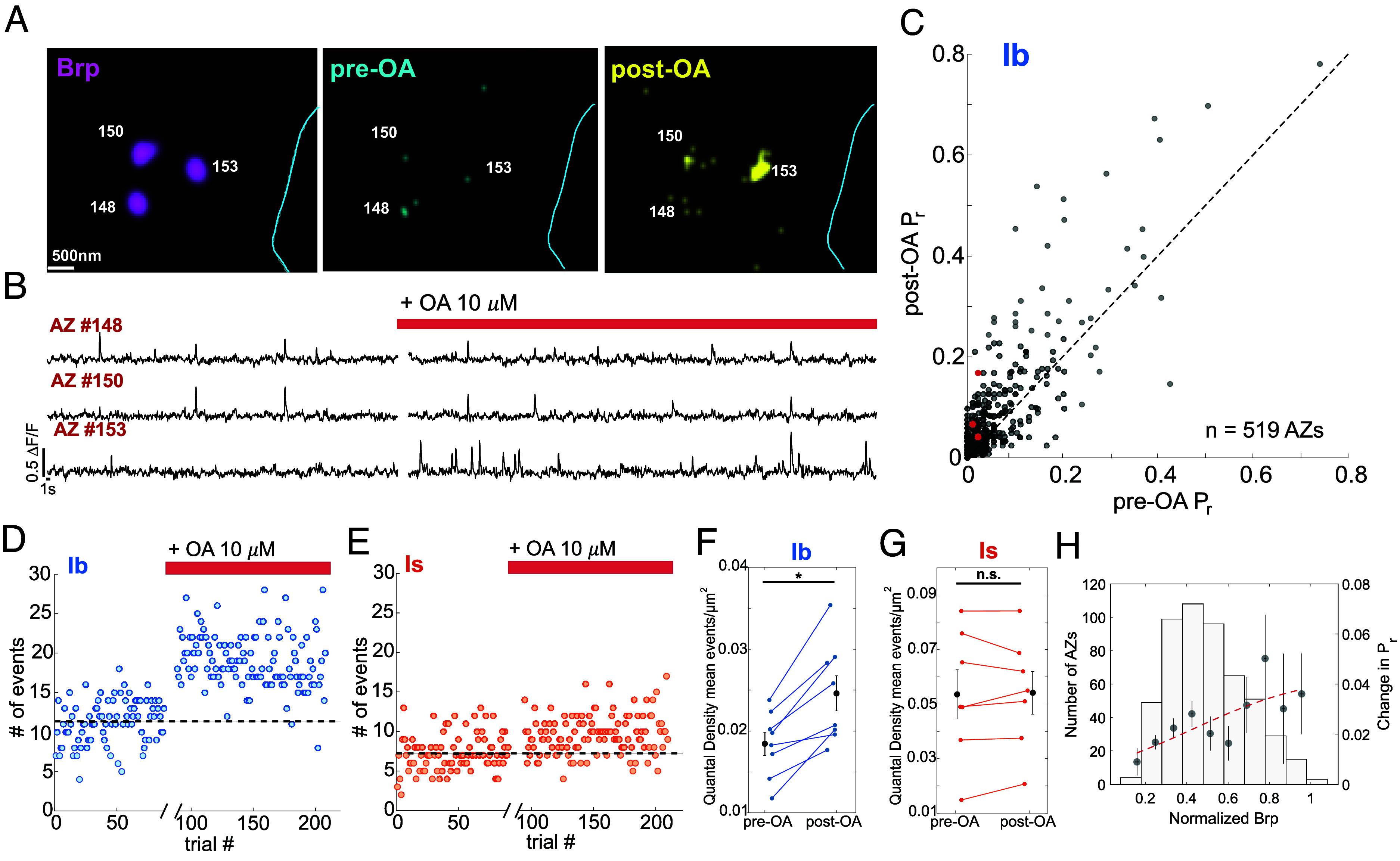
OA potentiates release from type Ib MN but not type Is MN. (*A*) *Left*: Example Ib bouton stained post hoc for the AZ scaffolding protein Bruchpilot (Brp, magenta). Each magenta punctum is an AZ. *Middle* and *Right*: Transmission event maps evoked by 0.2 Hz stimulation before addition of OA (*Middle*) and in presence of OA (*Right*). (Scale bar, 0.5 μm.) (*B*) SynapGCaMP6f ∆F/F recording of Ib MN glutamate transmission events from the three identified AZs in (*A*) before OA and 10 min after the addition of 10 μM OA (red bar). Quantal events were identified by fits to a single ΔF response template, with highly correlated pixels and frames flagged as active if they had ΔF/F amplitudes that were above a minimum threshold (typically between 0.04 and 0.05 ΔF/F) and at least 1.5 to 2 times larger than the SD of the values at that pixel. (See *Materials and Methods*). (*C*) Relationship between *P_r_* before and after OA for individual wildtype Ib MN AZs (n = 519 AZs from 4 animals, 1 NMJ each). The dashed black line is y = x. AZ#s 148, 150, and 153 from (*A*) and (*B*) shown as red dots. (*D* and *E*) Raw ∆F/F glutamate transmission events in response to MN nerve stimulation at 0.2 Hz during 10 min basal (pre-OA) period and in OA (OA: 10 μM) (post-OA; red bar), following a 10 min incubation period without stimulation (break in the *x*-axis). Ib MN (*D*) and Is MN (*E*), which innervate the same muscle, are imaged simultaneously. The dashed black line indicates mean number of events per trial before OA. (*F* and *G*) OA-induced change in quantal density (QD) (mean # of events per stimulus/μm^2^) for Ib MNs (n = 8 NMJs) (*F*) and Is MNs (n = 7 NMJs) (*G*) from the same muscle, except in one case where only Ib MN was imaged. Error bars are mean ± S.E.M. (* *P* < 0.05; n.s., not significant by the paired *t*-test). (*H*) Change in *P_r_* (post-OA *P_r_* – pre-OA *P_r_*) binned by normalized Brp amount. Vertical error bars are mean change in *P_r_* ± S.E.M. Horizontal error bars are mean normalized Brp ± S.E.M. The red dashed curve is sigmoidal fit to Change in *P_r_*: $y=0.04/(1+e^{-\left(x-0.43\right)*3.4}$). Sum of squared estimate of errors (S.S.E.) = 5.4 × 10^−4^.

To address this, we used spinning disk confocal microscopy to perform fast SynapGCaMP6f imaging to detect the component of the excitatory postsynaptic current carried by Ca^2+^ influx through postsynaptic GluRII receptors. As in our previous report (20), we applied an advanced optical quantal analysis technique, Quantal Synaptic Optical Reconstruction (QuaSOR), to enhance the spatial resolution of quantal transmission imaging, enabling assignment of release events to identified synapses, even in areas of dense packing (*SI Appendix*, Fig. S4*A*). To assess basal release, we measured action potential (AP)-evoked transmission at Ib/Is MN pairs that innervate muscle 4 during low frequency (0.2 Hz) motor nerve stimulation. We obtained a baseline recording of around 100 stimuli, stopped stimulation, incubated with 10 μM OA for 10 min, and then resumed stimulation for 100 more stimuli at 0.2 Hz in the continued presence of OA. We measured the total number of transmission events (∆F/F peaks time-locked to the stimulus) across the entire NMJ. We then followed up the functional imaging with antibody staining and Airy Confocal imaging of the presynaptic Bruchpilot (Brp), the *Drosophila* presynaptic scaffolding protein homolog of ELKS/CASK, which forms the characteristic AZ T-bar structure seen in electron microscopy (see Methods). Quantal imaging cumulative transmission maps aligned with the post hoc Brp maps revealed the probability of AP-evoked release (*P_r_*) at each of the dozens of identified AZs for each MN (Fig. 2 *A* and *B* and *SI Appendix*, Fig. S4 *A*–*C*).

In Ib MNs, OA increased *P_r_* across the population of AZs (Fig. 2 *B* and *C*). While the total number of transmission events per NMJ fluctuated from stimulus to stimulus, the potentiation of Ib glutamate release was stable for minutes (Fig. 2*D* and *SI Appendix*, Fig. S2 *A* and *C*), yielding an increase in QD (mean # of events per stimulus per square micrometer) (Fig. 2*F*). In Ib MNs, OA increased QD by ~40% (mean pre-OA QD = 0.018 ± 1.4 × 10^−3^ events/μm^2^, mean post-OA QD = 0.025 ± 2.2 × 10^−3^ events/μm^2^, *P =* 0.03). There was no effect of the DMSO carrier (*SI Appendix*, Fig. S3 *A* and *B*).

Synapses with similar basal *P_r_* varied widely in OA potentiation. For example, three neighboring AZs with similar basal *P_r_* (AZ #148: 0.02, AZ #150: 0.01, AZ #153: 0.02) (Fig. 2*B*) responded to OA with varying degrees of potentiation of *P_r_* (AZ # 148: 0.02 → 0.04, AZ #150: 0.01 → 0.06, AZ #153: 0.02 → 0.17) (Fig. 2 *B* and *C*). The differential potentiation at neighboring synapses suggests that the determinants of response to OA function locally at individual AZs. Across 519 AZs from 4 Ib NMJs (Fig. 2*C*), *P_r_* increased in 76% of the AZs and decreased in 23%, with 1% remaining unchanged.

We find that the more Brp there is at a synapse, the higher its OA potentiation (Fig. 2*H*, filled circles). While Brp explains some of the diversity in OA potentiation, AZs with similar Brp content showed different degrees of potentiation (Fig. 2 *A* and *B* and *SI Appendix*, Figs. S4 and S5), indicating that other determinants are involved. In striking contrast to the OA potentiation that we observed at synapses of type Ib MNs, type Is MNs, which had a ~threefold higher basal QD than did type Ib MNs, as shown previously (12, 20), showed no OA potentiation (mean pre-OA QD = 0.053 events/μm^2^, mean post-OA QD = 0.054 events/μm^2^) (Fig. 2 *E* and *G* and *SI Appendix*, Fig. S2 *B* and *D*). Together, these results show that OA potentiates glutamatergic transmission presynaptically and does so at Ib MNs but not Is MNs.

### OA Potentiation of Release Is Mediated by G_q_-Coupled OAMB Receptor and PLC.

**Fig. 3.**
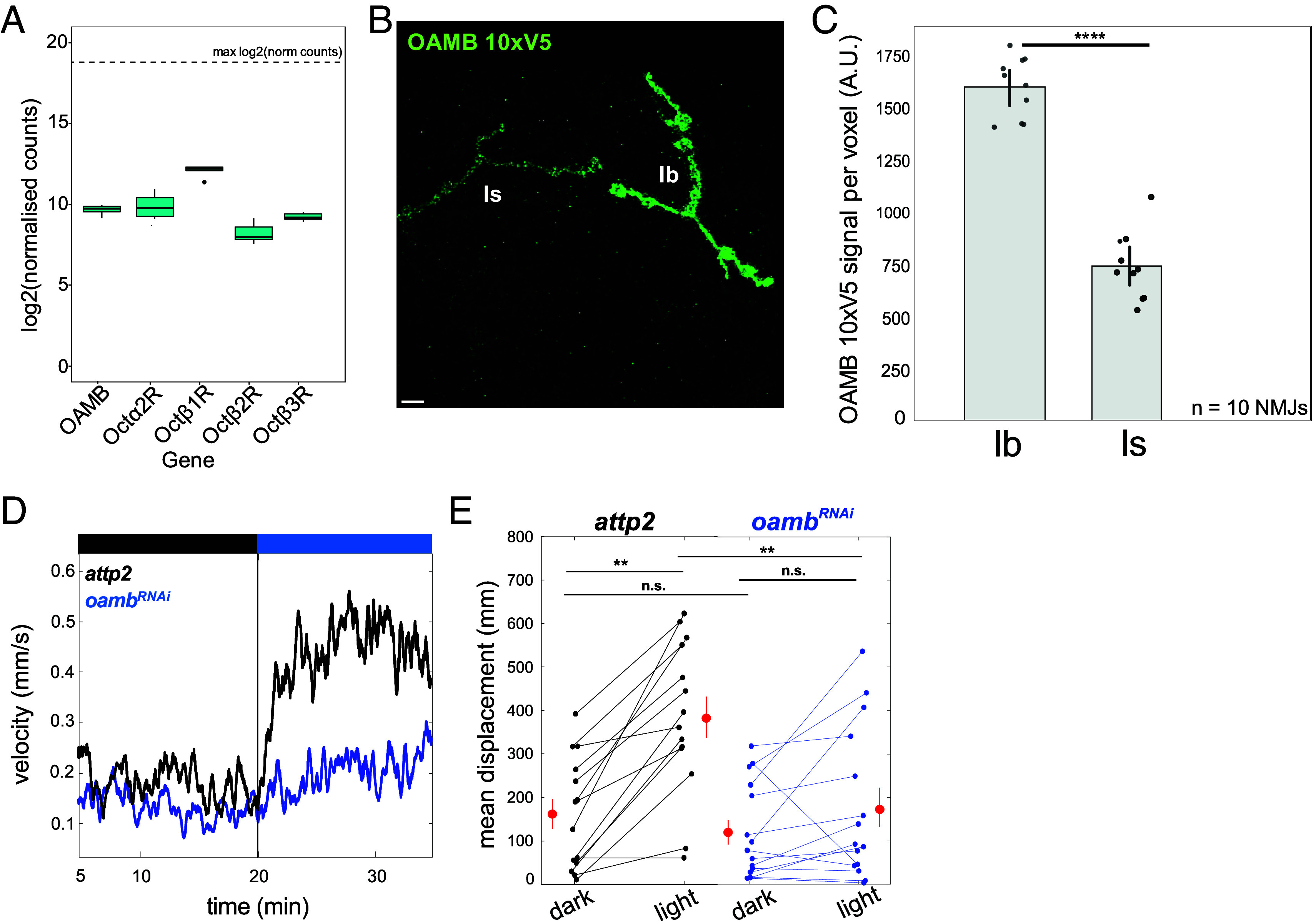
Type I MNs OA receptors include OAMB, which is enriched in the Ib MN and needed to boost locomotion. (*A*) RNAseq shows expression of five OA receptors in type I MNs. The dashed line shows the maximum normalized counts of any gene observed in *attp2* controls. Error bars are ± S.E.M (*B*) Example image of an NMJ showing a maximum projection confocal image of OAMB receptor staining (green) in a Ib and Is MN pair. Animals had the OAMB receptor CRISPR tagged with 10 copies of the V5 epitope. Staining was against the V5 epitope. (Scale bar, 5 μm.) (*C*) Bar graph shows the mean ± 95% CI of the mean OAMB staining levels (A.U. arbitrary units) per Ib and Is MNs. Each Ib and Is were paired on the same muscle (10 NMJs, 1 per animal). (**** *P* <0.0001 by the paired *t*-test). (*D*) Mean velocity (mm/s) of control larvae (*attp2*) (black, n = 15) and animals with OAMB knocked down in type I MNs (*oamb^RNAi^*; blue, n = 14) crawling in the dark (black bar) followed by under 505 nm light (blue bar). Recording starts at 5 min., following an adjustment period. (*E*) Mean total displacement for each animal in (*D*). *Attp2* (black, n = 15 animals), *oamb^RNAi^* (blue, n =14 animals). Red points are mean ± S.E.M. (*** *P* <0.001; ** *P* <0.01; n.s., not significant by Student’s paired *t*-test).

OA has three main classes of octopamine receptors (ORs) in *Drosophila*, which resemble adrenergic receptors: the β2-like G_s_-coupled Octβ1R, Octβ2R, and Octβ3R (5, 8, 30), the α2 -like G_i_-coupled Octα2R and the α1-like G_q_-coupled OAMB (8). To determine which, if any, of these is expressed in type I MNs, we examined our recent RNAseq dataset from type I MNs, which were FACS isolated by expression of a nuclear mCherry reporter under the type I MN-specific OK6-Gal4 line (31). We find that all five of the ORs are expressed in type I MNs (Fig. 3*A*), in agreement with a recent report from Littleton and coworkers (32).

**Fig. 4.**
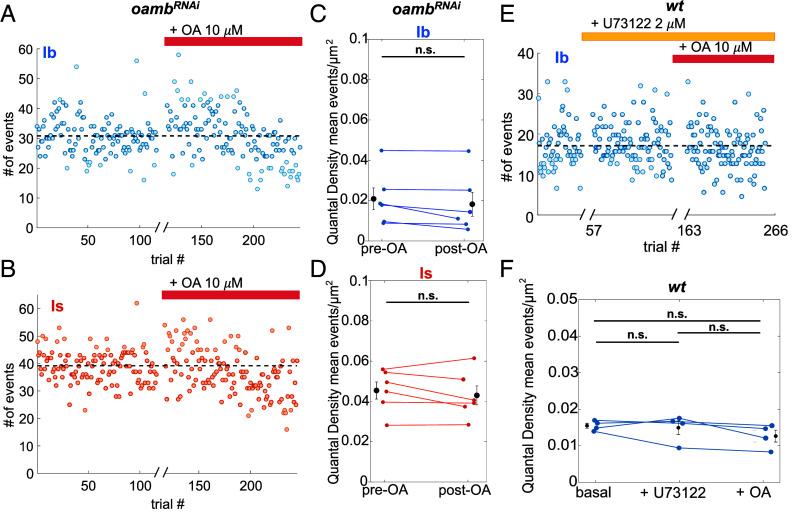
OA potentiation of release is mediated by the Gq-coupled OAMB receptor and PLC. (*A*–*D*) Knockdown of OAMB in type I MNs (*OK6>Gal4; UAS>oamb^RNAi^*) eliminates potentiation of release by 10 μM OA from type Ib MN synapses with no effect on Is MN synapses, which normally lack OA potentiation. (*A* and *B*) SynapGCaMP6f ∆F/F type I MN glutamate transmission events in response to nerve stimulation at 0.2 Hz during 10 min basal period, followed by a 10 min interval in presence of OA (no imaging, no stimulation, break in the *x*-axis), followed by 10 min of measurement of glutamate transmission in continued presence of OA. Raw glutamate transmission event counts per trial in response to stimulation at 0.2 Hz for an example Ib MN (*A*) and the Is MN (*B*) innervating the same muscle. Dashed black lines indicate mean number of events per trial before OA. (*C* and *D*) QD (mean # of events per trial/μm^2^) for Ib MN (*C*) and Is MN (*D*) pairs (n = 6 NMJs, 1 from each animal). Error bars are mean ± S.E.M. (n.s., not significant by the paired *t*-test). (*E* and *F*) Block of PLC eliminates OA potentiation. (*E*) Example *wt* Ib MN glutamate transmission events counts per trial, starting with 0.2 Hz stimulation during baseline period, switching to incubation with PLC blocker U73122 (no imaging, no stimulation, 10 min break in the *x*-axis), followed by 0.2 Hz stimulation in continued presence of U73122, followed by addition of OA (no imaging, no stimulation, 10 min break in the *x*-axis), followed by 0.2 Hz stimulation in the continued presence of U73122 + OA. (*F*) Summary of four *wt* NMJs, including that shown in (*E*), shows no significant change in QD in response to OA when preincubated with U73122. Points in black with error bars show mean QD ± S.E.M. (n.s., not significant by Student’s paired *t*-test).

Octβ1R, Octβ2R, and Octβ3R activate adenylate cyclase, and Octβ2R has been shown to promote axon outgrowth in both type I and type II MNs, thereby chronically increasing total type I MN glutamate release due to an increase in the number of synapses (32). However, a change in transmitter release probability has not been described for these receptors. Octα2R is G_i_-coupled and inhibits transmitter release (33). In contrast, the α1 adrenergic receptor, to which the *Drosophila* OAMB is related, is typically expressed presynaptically and boosts release (34), making it an attractive candidate mediator of presynaptic potentiation by OA. We tested this idea by selective RNAi knockdown of OAMB in type I MNs (*w^1118^*; OK6-Gal4/UAS-Dcr2; OAMB^RNAi^ /+). We found that the OAMB knockdown eliminated the OA-induced potentiation of glutamate release from type Ib MNs (Fig. 4 *A* and *C*) and had no effect on basal release. In the OAMB knockdown, as in wildtype animals (Fig. 2 *E* and *G*), release from type Is MN was not altered and remained insensitive to application of OA (Fig. 4 *B* and *D*). We wondered why OA modulates release from type Ib but not type Is MNs. Recent RNAseq analysis indicated that type Ib MNs express a higher level of OAMB transcript than do type Is MNs (32). To assess whether this results in different levels of OAMB protein in presynaptic nerve terminals, we CRISPR-tagged OAMB with ten tandem copies of the V5 epitope. Staining with anti-V5 antibody revealed strong staining in the mushroom body (*SI Appendix*, Fig. S6*A*), as expected for OAMB (6). In body wall muscle, expression of OAMB was ~twofold higher in the terminal axons of type Ib MNs than type Is MNs (mean Ib expression = 1592 ± 144 A.U. vs mean Is expression = 737 ± 161 A.U., n = 10 NMJs; *P* < 0.0001 by paired Student’s *t*-test) (Fig. 3 *B* and *C*). There was also no observed expression of OAMB in type II MNs (*SI Appendix*, Fig. S6*B*).

Strikingly, compared to *attP2* controls(mean locomotion in the dark = 162 ± 34 mm, mean locomotion in the light 384 ± 47 mm, n = 15 animals, *P* = 0.0007, paired Student’s *t*-test), knockdown of OAMB in type I MNs reduced the light-induced stimulation of locomotion and showed no baseline (in the dark) locomotor defects (mean locomotion in the dark = 120 ± 28 mm, mean locomotion in the light 177 ± 45 mm, n = 14 animals, not significant, paired Student’s *t*-test) (Fig. 3 *D* and *E*). This suggests that light increases type II MN activity and, consequently, OA release, and that the OA activates OAMB in type Ib MN axons thereby increasing glutamate release and boosting locomotion.

Since OAMB is G_q_-coupled, we turned our attention to its target phospholipase C (PLC). As above, we imaged responses to 50 baseline stimuli at 0.2 Hz, applied the PLC- selective inhibitor U73122 at 2 μM for 10 min, stimulated again for 100 stimuli at 0.2 Hz and then added 10 μM OA for 10 min after which we again stimulated for 100 stimuli at 0.2 Hz. U73122 prevented the OA-induced increase in the number of events per stimulus (Fig. 4*E*) and this could be seen across experiments as the absence of an OA-induced increase in QD (Fig. 4*F*). These observations suggest that OA potentiates glutamate release from type Ib MNs through the activation of OAMB and its G_q_ target PLC.

### Presynaptic OA Potentiation is Mediated by DAG Modulation of Unc13A.

**Fig. 5.**
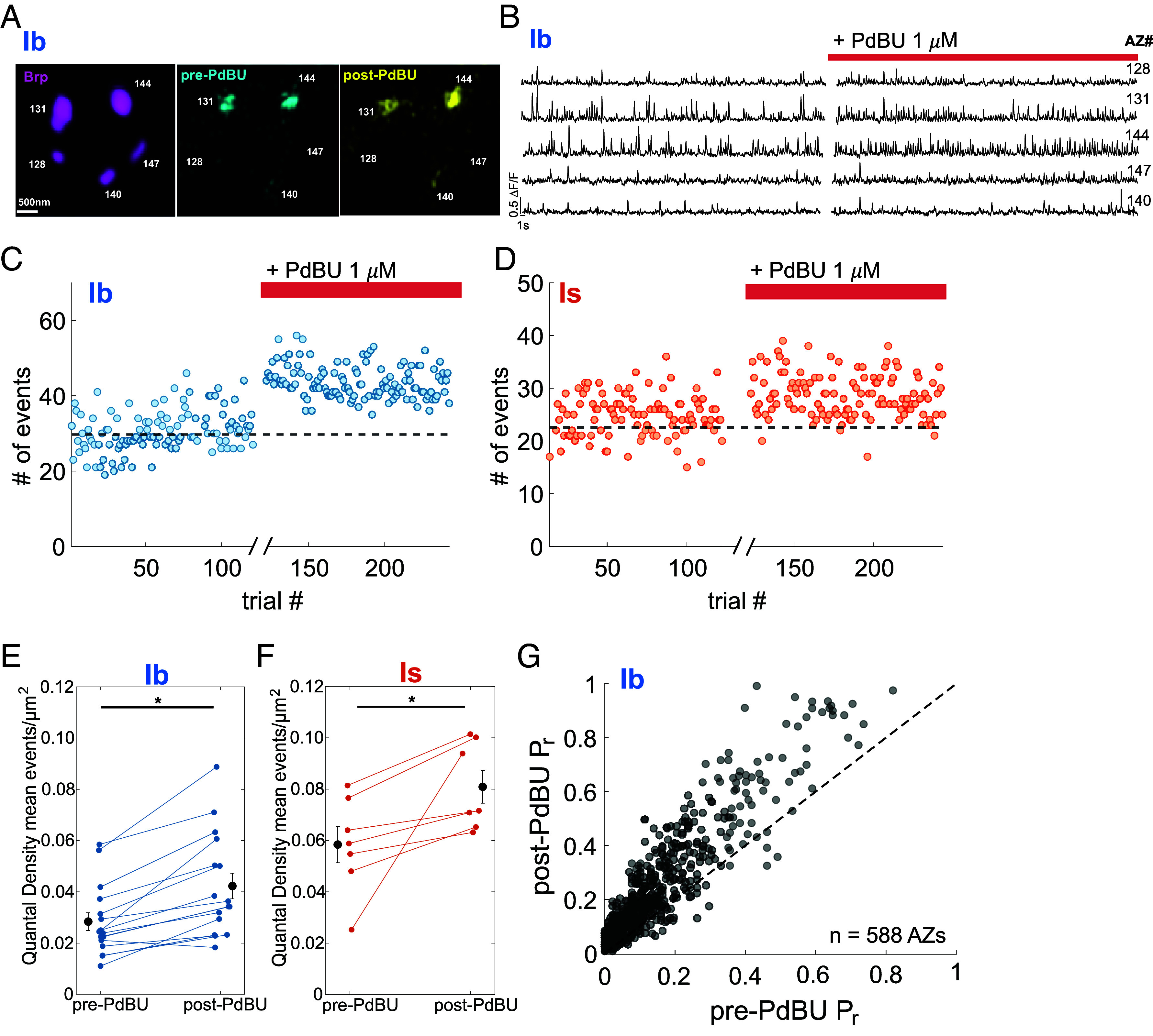
Membrane-permeable DAG mimic PdBU potentiates release from both type Ib and Is synapses. (*A*–*D*) 10 min baseline period followed 6 min interval (no imaging, no stimulation, addition of PdBU, *x*-axis break), followed by stimulation at 0.2 Hz stimulation in continued presence of PdBU. (*A*) Images of single Ib MN bouton showing AZ location (*Left*, Brp in magenta) in relation to glutamate transmission events evoked by 0.2 Hz stimulation in baseline period (*Middle*, cyan) and after addition of 1 μM PdBU (*Right*, yellow). (Scale bar, 500 nm.) (*B*) SynapGCaMP6f ∆F/F glutamate transmission events in response to MN nerve stimulation at 0.2 Hz for AZs shown in (*A*). Vertical (Scale bar, 0.5) ∆F/F. Horizontal (Scale bar, 1 s.) (*C* and *D*) Number of glutamate transmission events per stimulus over entire NMJ during stimulation at 0.2 Hz for an example muscle showing the Ib MN (*C*) and the Is MN (*D*). The dashed black line is the mean number of events per trial before PdBU. (*E* and *F*) Quantal densities (mean # of transmission events in entire NMJ per stimulus/μm^2^) for Ib MNs (n = 15 NMJs) (*E*) and Is MNs (n = 7 NMJs) (*F*), where each of the Is MNs was recorded simultaneously from muscle with shown Ib. Error bars are mean ± S.E.M. (* *P* < 0.05 by the paired *t*-test.) (*G*) Relationship between *P_r_* during baseline period (pre-PdBU) and following addition of PdBU (post-PdBU) for individual *wt* Ib MN AZs (n = 588, from 5 NMJs, 1 per animal, with AZ-matched QuaSOR analysis). The dashed black line is y = x.

PLC cleaves phosphatidylinositol 4,5-bisphosphate (PIP_2_) into inositol trisphosphate (IP_3_)—to release Ca^2+^ from intracellular stores—and DAG, both of which can promote transmitter release (27, 28). We tested the membrane-permeable DAG analog, phorbol-12,13-dibutyrate (PdBU), which has been shown to increase the excitatory postsynaptic current amplitude and the frequency of spontaneous release at the *Drosophila* larval NMJ (35). As seen with OA above, PdBU increased the type Ib synapse *P_r_* (Fig. 5 *A*, *B*, and *G*), increasing the number of events per AP for the entire type Ib NMJ (Fig. 5*C*) and increasing QD by ~60% (mean pre-PdBU QD = 0.028 ± 3.7 × 10^−3^ events/μm^2^, mean post-PdBU QD = 0.044 ± 5.1 × 10^−3^ events*/*μm^2^, n = 15 NMJs; *P* = 0.02, paired Student’s *t*-test) (Fig. 5*E*). Unlike what we observed with OA, PdBU also potentiated release from Is synapses (Fig. 5 *D* and *F*), suggesting that Is synapses contain the target of DAG but have lower expression of the OAMB receptor as confirmed here and in recent studies (32).

Similar to the potentiation by OA, PdBU boosted evoked release at synapses across the range of basal *P_r_*, (Fig. 5 *G*). Out of the 588 AZs in five type Ib NMJs, PdBU increased *P_r_* in 90.3% (n = 531 AZs) and decreased *P_r_* in 9.7% (n = 57 AZs), with an average *P_r_* increase of 57% (*P* = 3.7 × 10^−16^, two-sample Kolmogorov-Smirnov test). These observations show that the large majority of Ib synapses, across a wide range of basal presynaptic strength, are potentiated by a DAG analog. Also, as with OA, the degree of PdBU potentiation varied greatly between Ib synapses that had similar basal *P_r_* (Fig. 5*G*).

One of the mechanisms through which DAG modulates transmitter release is by binding to the presynaptic protein Unc13 (36), which is involved in the priming and localization of synaptic vesicles (37). *Drosophila* has two splice isoforms of Unc13: Unc13A and Unc13B, which differ in effect on transmitter release, and localize at different distances from the core of the AZ (38, 39). To investigate whether Unc13 is the relevant DAG target, we knocked down both of the isoforms of Unc13 selectively in type I MNs (*w^1118^*; OK6-Gal4/+; SynapGCaMP6f/UAS-Unc13^RNAi^). Consistent with earlier work (38), the knockdown of Unc13 greatly reduced (by ~80%) the basal number of transmission events (4.9 ± 0.19 events/trial for *unc13^RNAi^* (*SI Appendix*, Fig. S7*A*) vs. 26.8 ± 0.47 events/trial for *wt* (Fig. 5*C*). In the Unc13 knockdown, PdBU had a reduced effect on the number of transmission events/stimulus (*SI Appendix*, Fig. S7*A*), suggesting that Unc13 is, indeed, the target of modulation by PdBU.

**Fig. 6.**
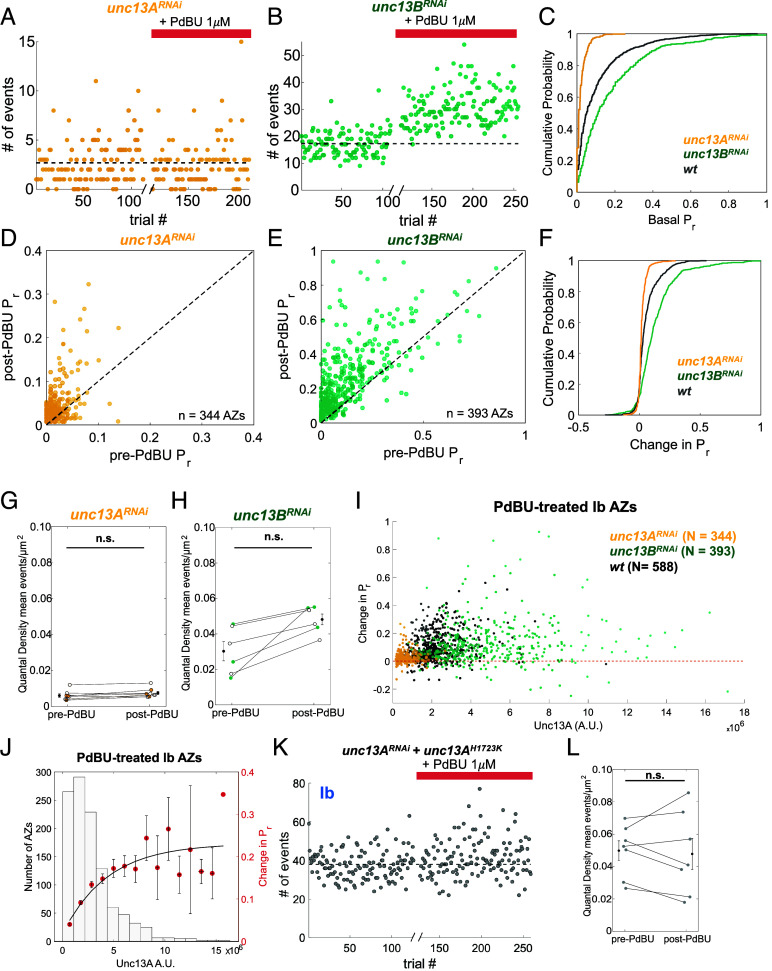
Unc13A is the PdBU target that boosts glutamate release in Ib synapses. (*A* and *B*) Raw event counts per trial to examine PdBU effect on example *unc13A^RNAi^* Ib MN (*A*) and *unc13B^RNAi^* Ib MN (*B*). Break in the *x*-axis marks addition of PdBU (1 μM) followed by a 10 min period of transmission imaging in continued presence of PdBU. The dashed black line is mean number of events per trial before PdBU. (*C*) Cumulative probability of basal *P_r_* distributions for individual Ib AZs. Basal *P_r_* distributions are significantly different by a two-sample Kolmogorov–Smirnov test: *p* = 3.78 × 10^−16^ for *wt* (gray, same AZs as in Fig. 5 *G*) vs *unc13B^RNAi^* (green), *P* = 9.31 × 10^−53^ for *wt* (gray) vs *unc13A^RNAi^* (yellow) and *P* = 4.19 × 10^−83^ for *unc13B^RNAi^* (green) vs *unc13A^RNAi^* (yellow). (*D* and *E*) Relation of basal *P_r_* (pre-PdBU) to PdBU-potentiated *P_r_* (post-PdBU) for individual Ib MN AZs in *unc13A^RNAi^* (n = 344, from 3 NMJs) (*D*) and *unc13B^RNAi^* Ib MN AZs (n = 393, from 3 NMJs) (*E*). The dashed black line is y = x. (*F*) Change in *P_r_*post-PdBU distributions for individual Ib AZs. Change in *P_r_* distributions are significantly different by a two-sample Kolmogorov–Smirnov test: *P* = 2.5 × 10^−4^ for *wt* vs *unc13B^RNAi^*, *P* = 4.97 × 10^−54^ for *wt* (gray) vs *unc13A^RNA i^*(yellow) and *P* = 5.54 × 10^−49^ for *unc13B^RNAi^* (green) vs *unc13A^RNAi^* (yellow). (*G* and *H*) QD for Ib MNs in *unc13A^RNAi^* animals (*G*; n = 8 NMJs) and *unc13B^RNAi^* animals (*H*; n = 6 NMJs) before and after addition of PdBU (1 μM). Black symbols with error bars are mean QD ± S.E.M. (n.s. is not significant; * *P* < 0.05 by Student’s paired *t*-test). (*I*) Scatter showing the relationship between change in *P_r_* (post-PdBU *P_r_* – pre-PdBU *P_r_*) and Unc13A amount in arbitrary units (A.U.) for individual AZs pooled from Ib MNs in three genotypes: *unc13A^RNAi^* (yellow) *unc13B^RNAi^* (green) and *wt* (black). The dashed line is y = 0, representing no change. (*J*) Unc13A-dependent change in *P_r_* induced by PdBU in potentiated synapses pooled from the three different genotypes (n = 1137 AZs from 11 NMJs: 5 *wt,* 3 *unc13B^RNAi^*, 3 *unc13A^RNAi^*). *Left* axis: number of AZs per bin. *Right* axis: change in *P_r_* (filled circles). Vertical error bars are mean *P_r_* ± SEM. Horizontal error bars are mean normalized Unc13A ± S.E.M. The red line is an exponential fit: $y=(0.21*(1+e^{-\left(x-2.2*10^{-7}\right)}$) + 0.01). S.S.E. = 0.032. (*K*) Raw event counts per trial for example *unc13A^RNAi^ + unc13A^H1723K^* Ib MN, where native Unc13A is knocked down by RNAi and an RNAi-proof Unc13A with a mutation at the DAG binding site is expressed in type I MNs. Note restoration of baseline event counts to normal levels. The dashed black line is the mean number of events per trial before PdBU. (*L*) QD for Ib MNs in *unc13A^RNAi^ + unc13A^H1723K^* animals before and after PdBU (1 μM) (n = 7 NMJs). Black symbols with error bars are mean QD ± S.E.M. (n.s. is not significant by Student’s paired *t*- test).

To further determine how the two different Unc13 isoforms contribute to modulation by DAG, we knocked them down individually in type I MNs. Knockdown of Unc13A severely impaired basal evoked release in Ib MNs, reducing mean *P_r_* by ~80% (Fig. 6 *A*, *C*, *D*, and *G*), consistent with the core role for Unc13A in action potential triggered release. In contrast, knockdown of Unc13B *augmented* basal evoked release in Ib MNs, increasing mean *P_r_* by ~80% (Fig. 6 *B*, *C*, *E*, and *H*). This augmentation led us to ask whether the knockdown of Unc13B affects the expression of Unc13A. Indeed, we found that Unc13A at Ib synapses increased by ~40% in the Unc13B knockdown (*SI Appendix*, Fig. S7 *B*–*F*), suggesting a compensatory mechanism. The average level of Brp across Ib synapses was unchanged by the knockdown of either Unc13A or Unc13B.

Knockdown of Unc13A reduced PdBU presynaptic potentiation at Ib synapses to the point that it was barely detected in *P_r_* measurement (~20% boost) and there was no effect detected on QD (Fig. 6 *D*, *F*, and *G*). In contrast, the Unc13B knockdown increased the PdBU boost of QD to ~70% and average *P_r_* to ~90%, more than observed in wildtype animals (Fig. 6 *E*, *F*, and *H*). Given the high degree of variability in potentiation that we observe between synapses, we wondered whether the degree of potentiation is related to the quantity of Unc13A at the AZ. We find that synapses with higher levels of Unc13A show greater potentiation by PdBU (Fig. 6 *I* and *J*).

To disentangle the effects on basal *P_r_* from the effects on potentiation, we replaced the native Unc13A with a version of Unc13A (H1723K) that has its DAG site mutated such that it is insensitive to modification and modulation by phorbol esters (40, 41). We did this by combining the RNAi knockdown of native Unc13A with overexpression of an RNAi-proof Unc13A(H1723K). In these animals (*w^1118^*; OK6-Gal4/UAS- Unc13A^H1723K^; SynapGCaMP6f/UAS-Unc13A^RNAi^), basal release from Ib MNs was normal, but PdBU had no potentiating effect (Fig. 6 *K* and *L*), confirming the interpretation that Unc13A is the target of the DAG mimic PdBU.

**Fig. 7.**
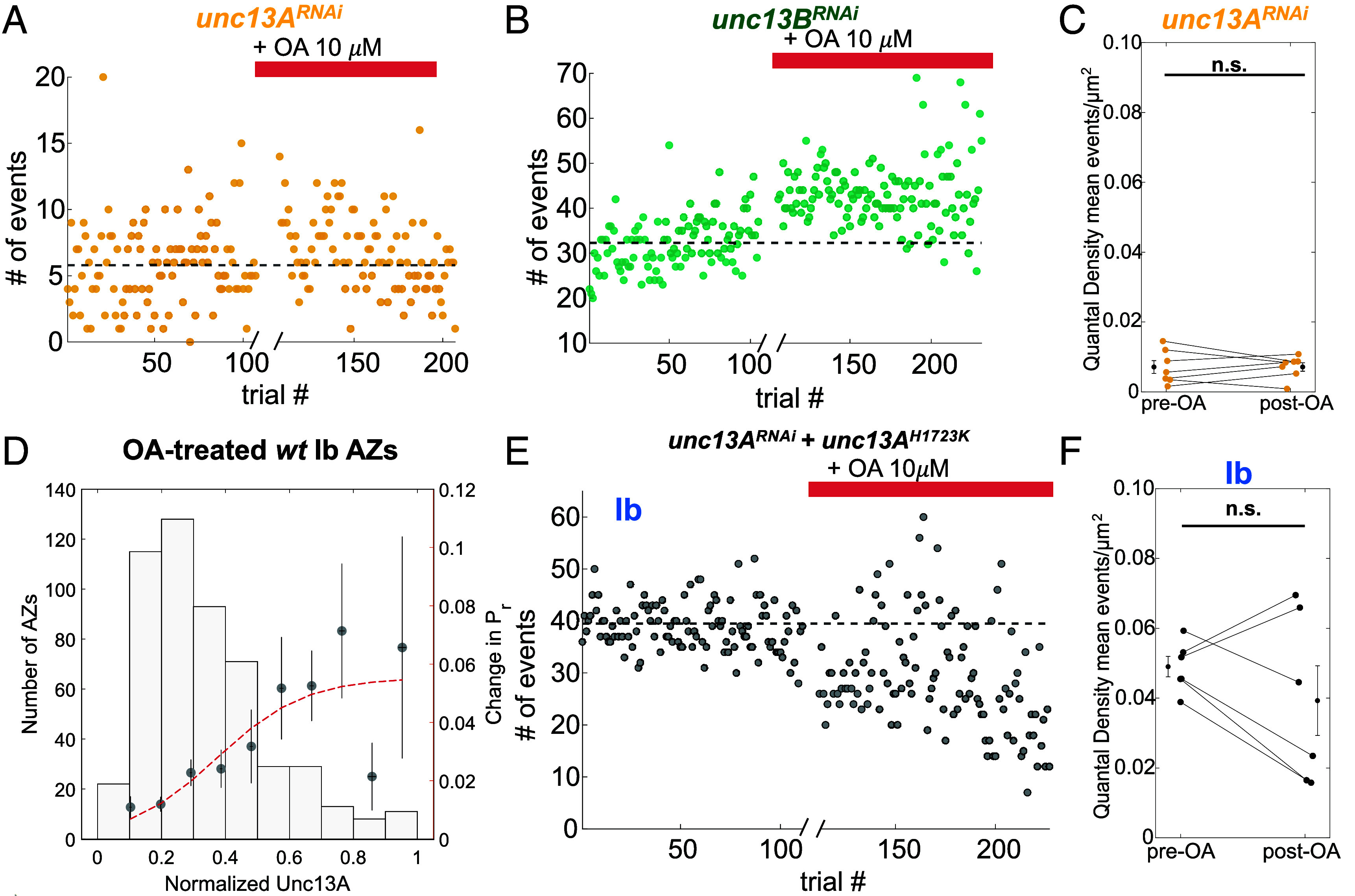
Potentiation by OA depends on presynaptic DAG modulation of Unc13A. (*A* and *B*) Raw event counts per trial for example *unc13A^RNAi^* Ib MN (*A*) and *unc13B^RNAi^* Ib MN (*B*). (*C*) QD for Ib MNs in *unc13A^RNAi^* animals before and after addition of OA (10 μM) (n = 7 NMJs). Error bars on the sides are mean QDs ± SEM. (n.s. is not significant by Student’s paired *t*- test). (*D*) Unc13A-dependent change in *P_r_* induced by OA. *Left* axis: number of AZs per bin. *Right* axis: change in *P_r_* (post-OA *P_r_* - pre-OA *P_r_*) filled circles. Vertical error bars are mean *P_r_* ± SEM. Horizontal error bars are mean normalized Unc13A ± SEM. (n = 588 AZs from 5 NMJs). The red line is sigmoidal fit: $y=0.05/(1+e^{-\left(x-0.37\right)*7.2}$). S.S.E. = 1.7 × 10^−3^. (*E*) Raw event counts per trial for an example *unc13A^RNAi^ + unc13A^H1723K^* Ib MN. The dashed black line is the mean number of events per trial before OA. (*F*) QD for Ib MNs in *unc13A^RNAi^ + unc13A^H1723K^* animals before and after OA (10 μM) (n = 6 NMJs). Black symbols with error bars on the sides are QDs ± S.E.M. (n.s. is not significant by Student’s paired *t*-test).

We then asked whether OA also acts through DAG modulation of Unc13A. We found that knockdown of Unc13A, eliminates OA-induced potentiation of release from Ib MNs (Fig. 7 *A* and *C*), but knockdown of Unc13B does not (Fig. 7*B*). Similarly to PdBU, we find that synapses with higher levels of Unc13A show greater potentiation both in response to OA (Fig. 7*D*). Furthermore, just as with PdBU, we found that replacing the native Unc13A with a version that is unable to bind DAG also eliminated OA-induced potentiation in Ib MNs (Fig. 7 *E* and *F*).

Based on the constellation of results, we conclude that OA potentiates release from Ib synapses by activating the G_q_-coupled OAMB receptor, which activates PLC, which in turn produces DAG that then binds to Unc13A in the AZ, thereby promoting glutamate release in proportion to the Unc13A content of that AZ.

**Fig. 8.**
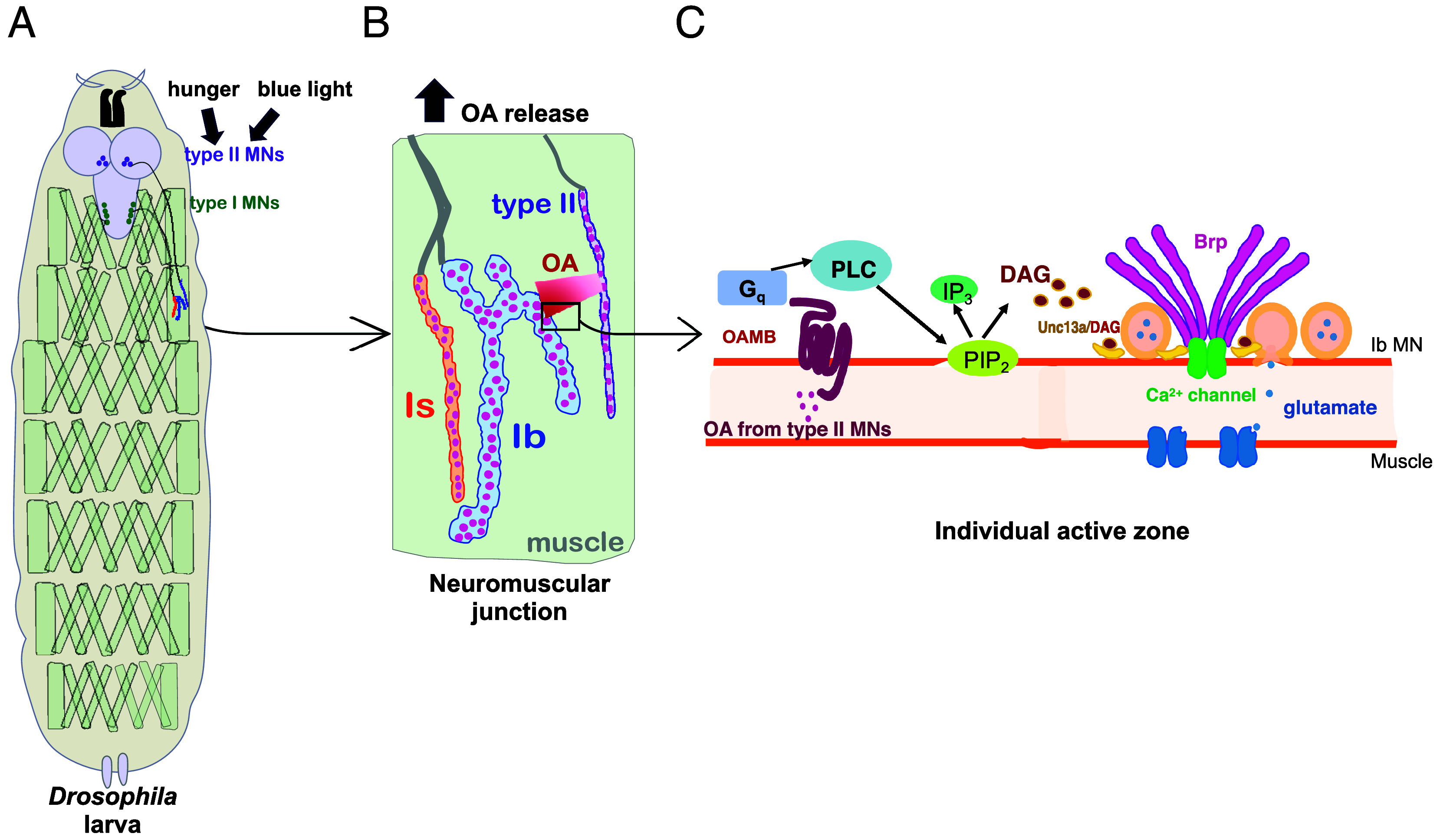
Model of peripheral catecholaminergic locomotion boost. (*A*) Hunger or blue light (and, likely, other signals) activate type II MN and boost locomotion (e.g. for foraging or escape) by switching the firing of octopaminergic type II MNs from low frequency tonic activity to high frequency bursts timed to precede and overlap with the locomotory bouts of glutamatergic transmission from type I MNs. (*B*) At the NMJ level, the boost in type II MN activity, described in (*A*), increases the release of OA from type II MNs. (*C*) At the individual AZ (synaptic) level, OA released from type II MN binds to the Gq-coupled OAMB (octopamine receptor) in type Ib MN presynaptic nerve terminals, activating phospholipase (PLC), which breaks down PI_4,5_P_2_ (PIP_2_) to generate IP_3_ and DAG. DAG binds to Unc13A and promotes its synaptic vesicle superpriming activity leading to increased release of glutamate and increased locomotion. OAMB expression is too low in type Is MN axons to support OA potentiation, resulting in potentiation of type Ib transmission and constancy of type Is transmission.

While OA boosts the probability of glutamate release from type Ib MNs, it has no effect on the nearby type Is MN (Fig. 4*B*). However, the AZs of Is MNs contain Unc13A and PdBU boosts release in Is synapses (Fig. 5*F*), indicating that they contain the capacity for DAG potentiation and suggesting that the difference between Ib and Is synapses may lie in the expression of OAMB. Consistent with this, OAMB is expressed at a lower level in Is MNs (Fig. 3 *B* and *C*). Together, our observations suggest that OA potentiation is mediated by the activation of a G_q_-coupled OA receptor on presynaptic Ib MN nerve terminals, which activates PLC to produce DAG, which binds Unc13A and boosts action potential evoked glutamate release (Fig. 8).

## Discussion

In *Drosophila,* OA regulates locomotion peripherally at the NMJs of body wall muscles, mediated by octopaminergic type II MNs (16, 22). Earlier work demonstrated that prolonged optogenetic stimulation of type II MNs stimulates the growth of type I and type II MNs axons, leading to increased locomotion (16). Additionally, hours of starvation were also found to increase locomotion, but only when type II MNs are intact (16). Our functional imaging directly demonstrates that hunger elevates type II MN firing frequency, shifting the pattern from tonic to bursting. Exposure to light triggers a rapid increase in locomotion (within 1 min), and this boost is blocked by optogenetic inhibition of type II MNs. This suggests that type II MNs also modulate locomotory drive on a fast time scale. Bursting activity of type II MNs coincides with or precedes the onset of glutamatergic transmission bouts by type Ib MNs, indicating that OA is released at the optimal time to modulate type I MN synaptic transmission and drive muscle contraction (12).

While OA has been shown to enhance the amplitude of fast glutamatergic EPSPs, the precise site of modulate—pre- or postsynaptic—was unclear (16). Our experiments reveal that this transmission boost occurs within minutes and is due to presynaptic potentiation of glutamate release from type Ib MNs and is mediated by OAMB, the G_q_-coupled OA receptor that we here, and others recently (32), found to be expressed in type I MNs. Consistent with G_q_ signaling, PLC, its product DAG, the known DAG target Unc13, and the Unc13 DAG binding site are all necessary for the modulation. Unc13 is involved in the priming and localization of synaptic vesicles (37) and DAG is thought to modulate Unc13 by displacing a water molecule at its C1-binding site, enabling synaptic vesicles to approach the plasma membrane in a process that “superprimes” them by lowering the energy barrier for synaptic fusion (42, 43).

In *Drosophila* there are two alternative splice products of Unc13, Unc13A, and Unc13B. Unc13A is located at the AZ, where it impacts AP-evoked release, whereas Unc13B is located peripherally, where it has little effect on evoked release (38). We find that knockdown of Unc13A, or its replacement with a mutant version lacking the DAG binding site, eliminates the potentiation. In contrast, knockdown of Unc13B leaves the potentiation intact. In fact, Unc13B knockdown increases Unc13A expression and boosts potentiation by the DAG-analog PdBU.

Thus, beyond the previously described type I MN growth effect of prolonged type II MN activity that was attributed to protein kinase A (16), OA released from type II MN activates presynaptic OAMB in type Ib MNs to elicit a rapid, DAG-dependent potentiation of the existing release machinery. The OAMB homolog of the mammalian brain, the α1 adrenergic receptor, has also been shown to enhance glutamate release, albeit through other signaling pathways (34).

Recent analysis has shown that even synapses made by one presynaptic neuron onto a single postsynaptic target can vary by an astonishing two orders of magnitude in basal *P_r_* (20). We find here that there is also a large diversity in presynaptic potentiation by OA among type Ib MN synapses, in both the level of Unc13A and the degree of potentiation varying over a range of ~10-fold. Since, as shown earlier (38) and confirmed here, *P_r_* is also positively correlated to the Unc13A content of the presynaptic AZ, presynaptically stronger type Ib synapses disproportionately contribute to catecholaminergic potentiation.

Strikingly, synapses of the type Is MN that innervate the same muscle are not potentiated by OA, even though they contain Unc13A and are potentiated by PdBU. This lack of OA potentiation could be explained by the lower expression of OAMB in type Is MNs. Type Is MNs differ from type Ib MNs in other respects as well: Their synapses have an approximately 3-fold higher *P_r_* and they lack presynaptic homeostatic plasticity (19). Together, these differences suggest that the type Is input is specialized for constancy and the type Ib input is designed for plasticity and modulation, with a subset of type Ib synapses contributing most strongly to catecholaminergic potentiation.

We conclude that a two-component presynaptic molecular mechanism—comprising an upstream OAMB receptor and downstream Unc13A promoter of transmitter release—determines whether synapses of glutamatergic MNs are subject to catecholaminergic modulation. Strong, OAMB-rich and Unc13A-rich synapses of tonic type Ib axons undergo potentiation, whereas weak, Unc13A-poor Ib synapses remain unaffected. Additionally, synapses of phasic type Is axons are not modulated, regardless of their basal *P_r_* and Unc13A content due to their low OAMB expression. The combined input and synaptic diversity provides the system with a balance between reliability and adaptability, enabling dynamic responses to changing behavioral conditions.

## Materials and Methods

### *Drosophila* Husbandry and Genetics.

Several flies were obtained from the Bloomington Drosophila Stock Center (BDSC) including; *unc13^RNAi^* (pVALIUM20 vector; inserted into attP2; BDSC line 29312), *oamb^RNAi^* (pVALIUM1 vector; inserted into attP2; BDSC line 31233), *attp2* (3rd chromosome attP docking site, BDSC line 36303), UAS-Dcr2 (BDSC line 24650), Tdc2-Gal4 (BDSC line 9313), Tdc2-Gal4.DBD(from the lab of Steve Stowers), 13XLexAop2-IVS-GCaMP6f-p10 (inserted into the attP5 site BDSC line 44277), and OK6 (BDSC line 64199. GtACR1 (20x-UAS-GtACR1.d.EYFP inserted into attP2) and Tdc2-lexA (two variants, one on the 3rd chromosome and one on the 2nd) were from the Liming Wang lab reported on previously (27). SynapGCaMP6f (3rd chromosome MHC-CD8-GCaMP6f-Sh) lines were reported previously (12, 20, 24), *unc13A^RNAi^* and *unc13B^RNAi^* were also reported on previously (38) and were obtained from the Sigrist lab. Flies were raised on standard corn meal and molasses media at 25 °C. A mix of male and female wandering third instar larvae was used in all experiments, and only actively crawling larvae were picked for experiments. When necessary, third instar larvae were screened for balancers. The balancers with larval markers included CyO^GFP^ (3xP3-EGFP variant) and Tm6B. All larvae contained a single copy of SynapGCaMP6f on the third chromosome. The following genotypes were used:

*wt* (*w^1118^*; +/+; SynapGCaMP6f/+)
*unc13^RNAi^* (*w^1118^*; OK6-Gal4/+; SynapGCaMP6f/UAS-Unc13^RNAi^)
*unc13A^RNAi^*(*w^1118^*; OK6-Gal4/+; SynapGCaMP6f/UAS-Unc13A^RNAi^)
*unc13B^RNAi^*(*w^1118^*; OK6-Gal4/+; SynapGCaMP6f/ UAS-Unc13B^RNAi^)
*unc13A^RNAi^ + unc13A^H1723K^*(*w^1118^*; OK6-Gal4/UAS- Unc13A^H1723K^; SynapGCaMP6f/UAS-Unc13A^RNAi^)
*oamb^RNAi^*(*w^1118^*; OK6-Gal4/UAS-Dcr2; SynapGCaMP6f/OAMB^RNAi^) or (*w^1118^*; OK6-Gal4/UAS-Dcr2; OAMB^RNAi^ /+) for locomotion tracking
*CRISPR tagged OAMB10xV5* (*w; +;* OAMB-10xV5)
*attP2* (*w^1118^*; R31C03-p65.AD/Tdc2-Gal4.DBD; attP2/+) for optogenetic experiments or (*w^1118^*; OK6-Gal4/UAS-Dcr2; attP2/+) for locomotion tracking
*GtACR1*(*w^1118^*; R31C03-p65.AD/Tdc2-Gal4.DBD; 20x-UAS-GtACR1.d.EYFP /+)
In vivo *imaging* cytosolic Type II GCaMP6f and SynapGCaMP6f: (*w^1118^;* 13XLexAop2-IVS-GCaMP6f-p10/Tdc2-lexA; MHC-CD8-GCaMP6f-Sh/+) or (*w^1118^;* 13XLexAop2-IVS-GCaMP6f-p10/+; MHC-CD8-GCaMP6f-Sh/Tdc2-lexA*)*
*GtACR1* (split Gal4): (*w^1118^*; Tdc2-Gal4/+; 20x-UAS-GtACR1.d.EYFP /+)

### CRISPR Tagging of OAMB.

CRISPR tagging of OAMB was performed by assembling a donor plasmid containing FLP recombinase target sites FRT and F3 flanking both sides of an inverted last coding exon common to OAMB isoforms RC, RD, RF, and RG. The FRT and F3 sites were oriented such that upon conditional expression of FLP recombinase a stable inversion would occur (44) that rearranges the final coding exon to the normal direction of transcription and allows expression of the V5-tagged variant of OAMB. The V5 tag was inserted between Valine 461 and Alanine 462 relative to the OAMB-RD protein isoform. The donor plasmid contained ~750 bp of sequence flanking the location of the guide RNAs. The sequence of the donor plasmid is available upon request. Two pCFD4 double guide RNA plasmids were assembled with pCFD4 OAMB Guide 1,2 containing guide RNA sequences AAGAGAAGGGGACGGGAA and CTGCGAGCGCGTGCACCA, and pCFD4 OAMB Guide 3,4 containing guide RNAs GCTGAGATAGCTACACTGAG and TACACTGAGGAAGCAATAGA. Genome-edited FRT-F3 OAMB-10XV5 flies were identified by taking single candidate males, crossing to a fly strain containing N-syb-GAL4 and UAS-FLP, followed by screening larval progeny by anti-V5 immunostaining. Germline inversion was accomplished by crossing FRT-F3 OAMB-10XV5 to nos-GAL4 and UAS-FLP to generate candidate germline inversion males, followed by single male crosses to a third chromosome balancer stock and larval anti-V5 immunostaining.

### UAS-Unc13A H1723K Expression Plasmid.

The UAS-Unc13A H1723K expression plasmid was made by making targeted mutations to the *Drosophila* melanogaster cDNA coding for Unc13A protein. The 8,616 base pairs were assembled as various gBlocks (IDT) using Gibson cloning into the JFRC2 plasmid. The H1723K mutation was selected, based on homology with rat and mouse *unc13*, to disrupt the PHNF motif; the C1-domain of Unc13 contains a zinc finger domain wherein the H > >K mutation disrupts phorbol ester binding (40, 41). To remove and fully supplant the endogenous fraction of Unc13A protein that would persist in being modified and regulated by phorbol esters, we “wobbled” specific regions in the cDNA to disrupt regions recognized by RNAi fragments yet still yielding an unaltered amino acid sequence. The goal in this endeavor was to allow concurrent expression of Unc13A H1723K protein alongside expression of the relevant RNAi against unc13A mRNA or a general RNAi to unc13 without isoform specificity. Targeted regions were selected and wobbled via use of a Python script, where base pairs were replaced with those of viable alternative codons typically by modifying the third base pair for each codon. This method of codon swapping is similar to codon optimization, and we replaced codons with preference to codons utilized by Drosophila melanogaster at a higher frequency (https://www.kazusa.or.jp/codon/cgi-bin/showcodon.cgi?species=7227). The RNAi for Unc13A expresses a short hairpin RNAi targeting base pairs 5’-TGGGTTAGGACATAATAATCTA-3’ (39). Thus, we modified this region to produce the resulting sequence: 5’-CGGCCTGGGCCACAACAACCTG-3’. Further, we wobbled the region corresponding to the RNAi line that is nonspecific toward elimination of unc13A and unc13B mRNA, Bloomington #29548, from the Harvard DRSC/TriP Functional Genomics Resources) consisted of a 484 base pair region we then modified corresponding to the unc13A cDNA region beginning at 5’-GACCGCATTAAGGTTCGTGT and ending at GTCCTGGGGTACCAGCTGTA-3’. The resulting protein sequence for Unc13A H1723K was checked via ClustalW (45) to confirm the occurrence of the singular H1723K mutation. Finally, the JFRC2 UAS-Unc13A H1723K was injected and introduced into attP1 landing site and successful integration was determined via expression of miniwhite in a white-null background (BestGene).

### RNA-Sequencing.

FACS sorting RNAseq and analysis described in ref. 31.

### Locomotion Analysis During Optogenetic Inhibition of Type II MNs.

Optogenetic behavioral experiments were performed within a plexiglass box staged inside an isolated dark chamber at 21 °C. Larvae were reared in the dark on molasses media supplemented with 1% 100 mM all-trans-retinal. Individual third instar larvae were placed within a covered yet ventilated 35 mm dish containing 3% agarose gel. Care was taken to pour the agar gel high enough to minimize the displacement of the larva along the sides of the dish in the z-dimension. Simultaneous behavioral activity of 6 larvae within 6 dishes was recorded with a CCD camera (fire-i 780b, Unibrain, 1 fps), which was backlit by an array of infrared LEDs. The behavioral assay consisted of 5 min of dark acclimation to the behavioral arena, followed by 15 min of crawling activity in the dark, then 15 min of crawling activity in under 505 mm LED illumination (1.135 mW/cm^2^ [without infrared (IR)], 1.1739 mW/cm^2^ [with IR)]. This dark-light cycle was then repeated for the group of 6 larvae resulting in two periods of dark treatment interspersed by two periods of light treatment. Behavioral analysis was performed using custom MATLAB routines where each larva’s displacement was determined using a 2D centroid tracking algorithm. To minimize noise in the crawling trajectory, centroids within 1 mm of each other over a 2 s interval were merged by calculating their center of mass.

### In Vivo Larval Imaging.

In vivo recordings of intact larval GCaMP6f and SynapGCaMP6f were conducted similarly to our previous report (12) (*SI Appendix*).

### Pharmacology.

Drugs were diluted according to the manufacturer’s instructions and stored at −20 °C in small, 20 μL aliquots. Dilutions were made the day of the experiment by thawing the aliquot and diluting it in high-Ca^2+^ HL3. For experiments involving acute application of pharmacological agents the following drugs were used at stated final concentrations: OA (Alfa Aesar, Lot # Z07E021) 2 mM stock diluted into 10 μM final concentration; PdBU (Phorbol 12,13-dibutyrate, Sigma Aldrich, Catalog # 761028, batch MKCJ2395) 2 mM stock diluted into 1 μM final concentration and PLC inhibitor (U73112 Abcam, Lot # GR3316421-23) 2 mM stock diluted into 2 μM final concentration. Each aliquot only underwent the freeze-thaw cycle once.

### SynapGCaMP6f Functional Imaging.

Optical quantal imaging was performed similarly to our previous reports (12, 20) (*SI Appendix*).

### Functional Registration and Bleach Correction.

The initial quantal image analysis was performed using custom-written MATLAB protocols, same as in our previous work (20) (*SI Appendix*).

### Quantal Event Detection.

To identify all quantal responses, we fit a single ΔF response template to the average temporal profile for all evoked responses at that NMJ, as described earlier (20) (*SI Appendix*).

### Quantal Synaptic Optical Reconstruction (QuaSOR).

After event detection, isolation, and verification, we then proceeded to analyze the 2D response profile of each event’s maximum ΔF/F spatial profile using the custom QuaSOR algorithm as in our previous work (20) (*SI Appendix*).

### QuaSOR Quantification.

Local QuaSOR synapse alignments were performed by identifying maximum evoked coordinate density positions for synaptic ROIs within a 350 nm radius. Maintaining their relative organization to nearby events, these QuaSOR event coordinates were averaged together with other synapses to generate a mean density image for synapse groupings. On its own, in absence of molecular imaging of AZ locations, synapse assignment in QuaSOR is not certain and could merge transmission events from neighboring synapses in regions of high synaptic density. We, therefore, focused our study on NMJs where QuaSOR maps were related to molecular structural imaging maps generated in Airyscan confocal imaging. Linear uniform global transformations in the x and y axes were applied to adjust for stretch of the tissue that occurred after the live optical quantal imaging during fixation and mounting on slides under cover glass for molecular imaging. The result yielded images in which QuaSOR transmission sites could be matched to Airy AZs. Single synapse AP-evoked transmission probability (*P_r_)* was determined by dividing evoked event counts within the single AZ domain by the number of motor nerve stimuli either pre- or postdrug (*P_r_* = events per synapses/# of stimuli; ranging from 0 to 1).

### Antibodies and Immunohistochemistry.

Larvae were fixed in room temperature Bouin’s fixative (Ricca Chemical Company, Arlington, TX) for 5 to 6 min, permeabilized in PBS with 0.1% Triton X100 (PBT) and blocked in PBS with 0.1% Triton X100, 2.5% normal goat serum, and 0.02% sodium azide (PBN). All antibody incubations were performed in PBN and all washes were performed in PBT. Mouse anti-Brp (nc82; Developmental Studies Hybridoma Bank, Iowa City, IA) was used at 1:100 for Airyscan imaging. Chicken anti-GFP (Thermo Fisher A10262; Thermo Fisher Scientific Waltham, MA) was used at 1:1,000 to label SynapGCaMP6f in fixed samples. Rabbit anti-Unc13A antibody (Sigrist lab) was used at 1:1,000. For OAMB imaging, rat anti-V5 epitope (Novus NBP2-81037) was used at 1:500. Alexa Fluor 488 goat anti-chicken (Thermo Fisher A11039), Alexa Fluor 488 donkey anti-rat (Invitrogen A48272), Alexa Fluor 555 goat anti-rabbit (Thermo Fisher A32727), and Alexa Fluor 647 goat anti-mouse (Thermo Fisher A21235) secondary antibodies were all used at 1:1,000. Antibodies obtained from the Developmental Studies Hybridoma Bank were developed under the auspices of the National Institute of Child Health and Human Development of the NIH and maintained by the Department of Biological Sciences of the University of Iowa, Iowa City, IA. We confirmed the specificity of the Unc13A antibody by comparing staining in control animals and in *unc13A^RNAi^* animals. To obtain uniformity in antibody staining, we fixed and stained the different genotypes compared within an experiment simultaneously with the same reagents.

### Confocal and Airyscan Imaging and Analysis.

Following antibody incubations and washes, larval fillets were mounted in Vectashield (H-1000; Vector Laboratories, Burlingame, CA) or Vectashield HardSet (H-1400). Airyscan imaging was performed on a Zeiss LSM 980 microscope. All samples were imaged with a 63X oil immersion objective (NA 1.4, DIC; Zeiss) using Zen software (Zeiss Zen black 2.3 SP1). All imaging data were collected using identical imaging and processing parameters for a given experiment set. Unless otherwise noted all Airyscan images are displayed as Gaussian filtered maximum intensity projections that were generated using custom-written MATLAB routines. Airyscan imaging data were acquired using a tiling strategy, whereby smaller volumes of each NMJ were acquired sequentially and stitched together. Brp reconstructions for matching to QuaSOR data were acquired on the LSM 880 system. Briefly, each imaging volume was acquired with an additional magnification of 4x with a 5 AU pinhole, 2 μs pixel dwell times, an x-y dimension of 1024 by 1024 px (processed to 1,000 by 1,000 px) at 11 nm/px, and axial z spacing of 159 nm. Each of the three-channel volumes (anti-GFP/Alexa 488, anti-Brp/Alexa 647, and anti-Unc13A/Alexa 555) were scanned sequentially.

Airyscan processing of all channels and z slices was performed in Zen (Zeiss Zen Black v2.3 SP1) using superresolution settings. We then sequentially stitched each 3D volume together in Fiji (NIH ImageJ Version 2.0.0-rc-43/1.52n) using pairwise stitching with linear blending. Alignments were performed to maximum intensity pixels of Brp puncta in overlapping volumes. AZ locations in stitched Airyscan datasets were calculated by masking the volumetric Brp data. Sites were initially identified using a local 3D Brp intensity maxima with a minimum distance of 150 nm from neighboring maxima. AZ identifications were manually validated and corrected when neighboring sites were misidentified. AZ-specific Brp voxel intensities were calculated by identifying 3D- connected voxels to each AZ maxima for isolated AZs. To avoid artifacts of stitching and bleaching of overlapping regions, pixel quantifications were collected using the original image pixel intensity information, prior to stitching, with intensity values calculated from 3D voxel AZ masks.

### Structure–Function Matching.

QuaSOR data were matched to the corresponding Airyscan data. First, the two datasets were roughly aligned by rotating either the QuaSOR or the Airyscan data to match the orientation of the corresponding map. Once roughly aligned, sites were matched to one another in a pairwise fashion taking care to match each Airyscan AZ to a corresponding location on the QuaSOR map. We used low-density areas to match all unambiguous AZ pairs with clear relative orientations and positions. Following these areas, we used the relative positioning of AZs around these pairs to match the denser regions or match AZs with no corresponding QuaSOR activity to an empty region of the QuaSOR map. In the Airyscan matched data, we sorted all QuaSOR event coordinates to their nearest QuaSOR AZ centroid location. All pairs had to minimize the local alignment variance within each bouton as determined by the alignment vector between sites. The quality of the AZ matching was further confirmed by using the paired coordinate positions to generate vectors to transform the QuaSOR coordinates into the matching Airyscan pixel-space. This was done by first converting the relative QuaSOR coordinates into a matched pixel-space image and then using a 2D, locally weighted, mean transformation method, with groupings of 8 to 14 AZs being used as control points in the local weighting. Successful site pairing generated accurate remapping of QuaSOR events onto the appropriate AZ for either structural imaging technique. However, all quantifications were performed on untranslated data in order to eliminate artifacts of transformation.

### Statistics and Reproducibility.

Student’s paired t-test was used to compare type Ib MN QD values measured before drug to after drug addition. Two-sample two-sided Kolmogorov–Smirnov tests was used to compare pooled cumulative distributions. One-way ANOVAs NMJ Ib properties for distributions of data between multiple bins. Unless otherwise noted, reported values are mean ± SEM. Sigmoidal and exponential fits were done using custom MATLAB code using nonlinear optimization using a multistart algorithm to obtain the best fit. The goodness of fit was assessed using the sum squared error of the residuals.

The specific statistical tests as well as the number of replicates including numbers of animals, NMJs, or AZs are all provided in the corresponding figures and/or figure legends. For all figures, significance markers are *P < 0.05, **P < 0.01, ***P < 0.001, or n.s. not significant for the comparisons indicated in the figure or figure legend. Representative QuaSOR and Airyscan images presented in figures were all reproduced in multiple animals. To assess reproducibility, recordings were done on only one NMJ per larva (i.e., the number of NMJs = the number of larvae) in recordings that were performed within 40 min of the beginning of the dissection, thereby ensuring health and similar conditions.

## Supplementary Material

Appendix 01 (PDF)

Movie S1. **Tracking of Ib and type II MNs. Left:** Example of live imaging of SynapGCaMP6f in type Ib and cytosolic GCaMP6f in type II MN. **Right:** Activity traces from the video on the left. Type II MN(orange trace, top right) is either in the ON or OFF state. Type Ib MN (blue trace, bottom right) shows the change in the ∆F/F over time.

## Data Availability

Source data are provided as a source data file and will be deposited in the Figshare repository (46). All other data are included in the manuscript and/or supporting information. Code used for trace extraction, live larva tracking, and analysis pipeline will be available in a Github repository at https://github.com/isacofflab upon publication (47). QuaSOR custom code is available in the GitHub repository under the filename: https://github.com/newmanza/Newman_QuaSOR_2021 https://doi.org/10.5281/zenodo.5711302 (48).
