# Supplementary material for: Synapse-specific catecholaminergic modulation of neuronal glutamate release
Source: Proc Natl Acad Sci U S A. 2024 Dec 30;122(1):e2420496121. doi: 10.1073/pnas.2420496121 (PMC11725921; doi:10.1073/pnas.2420496121)
Supplement: Supplementary file 1 — Appendix 01 (PDF) [file pnas.2420496121.sapp.pdf]

Supplementary Information

Supplementary Methods

In vivo larval imaging

In vivo recordings of intact larval GCaMP6f and synapGCaMP6f were conducted similarly to our previous report[12] (see Supplementary Information). Briefly, larvae were placed into a custom-made PDMS chamber with internal dimensions of 500 µm depth and 1 mm width (Supplementary Fig. 1a). Larvae were oriented dorsal side up within the chamber and gently sealed using standard coverslip glass. After a 3-minute acclimation period, imaging was performed with an Axio Zoom.V16 microscope (Zeiss), equipped with a 2.3× 0.57 NA objective, an FS 38 HE filter set (Zeiss; excitation BP 470/40nm HE; emission 525/50nm HE), and a Photometrics PRIME 95B camera. Image acquisition was performed on Micro-manager. Anterior half of the whole larva or dorsal muscles 1, 2, 9, and 10 from segments A1, A2, or A3 were used. These anterior segments provided the clearest images without significant contamination from the autofluorescent gut, additionally, larvae were raised on yeast-supplemented Carolina Instant Drosophila Medium Formula 4-24 to reduce the gut autofluorescence. For image acquisition, 50 ms images were continuously acquired at 20 Hz frequency for 10 minutes with a final magnification of 260X and a 2 µm depth of field (aperture fully opened). The large depth of field prevents the NMJs from moving out of focus during small movements. All MN activity was endogenous and data were only used from larvae that exhibited sustained activity following the mounting and imaging process. The analysis excluded recordings of NMJs where there was too much movement or that moved completely out of focus.

SynapGCaMP6f functional imaging

Optical quantal imaging was performed similarly to our previous reports[12,20]. To summarize, third instar larvae were dissected on PDMS (Sylgard 184, Dow Corning, Auburn, MI) pads in ice-cold HL3 solution containing, in mM: 70 NaCl, 5 KCl, 0.45 $CaCl_2$, 20 $MgCl_2$, 10 $NaHCO_3$, 5 trehalose, 115 sucrose, 5 HEPES, and with pH adjusted to 7.2. Following removal of the brain and the VNC, larval fillets were washed and imaged in room temperature HL3 containing 1.5mM Ca^{2+} and 25mM Mg^{2+}. Fluorescence images were acquired at room temperature with a Vivo Spinning Disk Confocal microscope (3i Intelligent Imaging Innovations, Denver, CO), using a 63 × 1.0NA water immersion objective (Zeiss), 1.2X optical adapter, LaserStack 488 nm (50 mW) laser, CSU-X1 A1 spinning disk (Yokogawa Tokyo, Japan), standard GFP filter, and EMCCD camera (Photometrics Evolve512, Tucson, AZ). All live SynapGCaMP6f imaging recordings were done on ventral

longitudinal abdominal muscle 4 at segments A3-A5 of third instar larvae. All imaging was performed using 50 ms exposures (20 fps) of the full camera sensor (512 × 512 px).

Nerve stimulation was performed with a suction electrode attached to a Stimulus Isolation Unit (SIU, ISO-Flex, A.M.P.I. Jerusalem, Israel), with 100 us stimulus duration. The intensity of the stimulus was adjusted to recruit both Ib and Is axons (verified through imaging) and kept constant throughout the imaging session. Nerve stimulation and imaging were synchronized using custom-written Matlab scripts (Matlab Version 2015b, MathWorks, Inc., Natick, MA) in order to control the SIU and trigger imaging episodes with SlideBook (v6.0.16, 3i Intelligent Imaging Innovations).

The basal evoked transmission protocol was the following: at each NMJ we collected action potential (AP)-evoked responses during short episodes in which a single stimulus was delivered. The frequency of stimulation was 0.2 Hz. Each episode consisted of 10 frames (50 ms exposure) with 3-4 baseline frames prior to nerve stimulation around frame 5. There was a minimum of 100 episodes collected pre-drug exposure and a minimum of 100 episodes collected post-drug exposure. For the experiments where the PLC inhibitor was added (Fig. 4e,f) we first collected a 50 episodes to ensure a steady baseline, applied the PLC inhibitor and waited for 10 minutes before collecting another 100 episodes. Afterwards, we added OA, waited 10 minutes and collected at least another 100 episodes. Minor focusing adjustments were made between trials if deemed necessary.

For comparability between experiments, recordings were done on only one NMJ (Ib-Is pair) per larva and recordings were performed within 40 minutes from the start of the dissection, ensuring the animals health. As in our previous report[20], we alternated which segments (A3-A5) we imaged from since no significant difference was found between segment.

Functional registration and bleach correction

The initial quantal image analysis was performed using custom-written MATLAB protocols, same as in our previous work[20]. Individual stimulus episodes were excluded due to out of focus NMJs, moving NMJs or failed axon recruitment. Otherwise, all movies were filtered (Gaussian low-pass filter), to reduce high-frequency noise. Image analysis areas were then separated into Ib and Is NMJ regions. All imaging data were registered using a multi-stage approach, during which all images were registered to a common reference image (usually the first frame of the first image), even when multiple treatment protocols were acquired (pre- and post-drug).

Following area selection and reference image selection, NMJs were tracked relative to this reference image using a rigid subpixel registration method to remove any large movements within

the NMJ imaging area[20,24,46]. As in our previous work[20], we corrected for local bouton movements using a custom diffeomorphic implementation of a demons algorithm[47] Once motion corrected, movies were bleach corrected using a fit for a double exponential bleach correction curve to the mean baseline pre- and post-stimulus fluorescence data for each trial separately. Following bleach correction, both ΔF and ΔF/F movies were generated using the first image as the baseline fluorescence (F0) image for the episodic data.

Quantal event detection

To identify all quantal responses, we fit a single ΔF response template to the average temporal profile for all evoked responses at that NMJ. As in our previous report[20], each pixel's temporal response was analyzed independently to determine if it had a high degree of correlation with the template response, as determined by the degree of cross-covariance. These highly correlated pixels and frames were then flagged as active if they had ΔF/F amplitudes that were above a minimum threshold (typically between 0.04 and 0.05 ΔF/F) and at least 1.5–2 times larger than the standard deviation of the values at that pixel. We also maintained a minimum allowable time between subsequent events at each pixel (100 ms) to prevent over-fitting. Coactive pixels were then grouped together into a single response field. This produced a single, isolated, maximal temporal projection image of the response's spatial profile. To eliminate false positives, we applied size, shape, and amplitude thresholds to these response fields. Following detection processing, events were sorted into the appropriate category using the timing of the response relative to the stimulus timing. Only a 200 ms window, following each AP, was used to assign evoked responses.

Quantal synaptic optical reconstruction (QuaSOR)

After event detection, isolation, and verification, we then proceeded to analyze the 2D response profile of each event's maximum ΔF/F spatial profile using the custom QuaSOR algorithm as in our previous work[20]. Briefly, we first looked at all the identified responses from our quantal detection and isolated small ROIs containing individual or small groups of partially overlapping response fields that corresponded to individual or small groups of events. These smaller ROIs were then subjected to independent 2D Gaussian mixture model fitting of the isolated ΔF/F spatial profiles. Following 2D Gaussian mixture model fitting for all response ROIs, all event functions were then remapped onto a common coordinate space and merged to define a single set of 2D Gaussian functions for each quantal response. The peak positions of each 2D Gaussian component were used to define event locations in a 21.2 nm x 21.2 nm pixel coordinate space. For visualization purposes, maps were generated by applying a normalized 2D Gaussian filter to each event coordinate prior to adding each event to the overall image. In this way each pixel contains an approximation of the event density at that location. To facilitate AZ matching we sometimes

reoriented the QuaSOR data by rotating and translating all the coordinates prior to re-rendering the maps to match the structural Airyscan data.

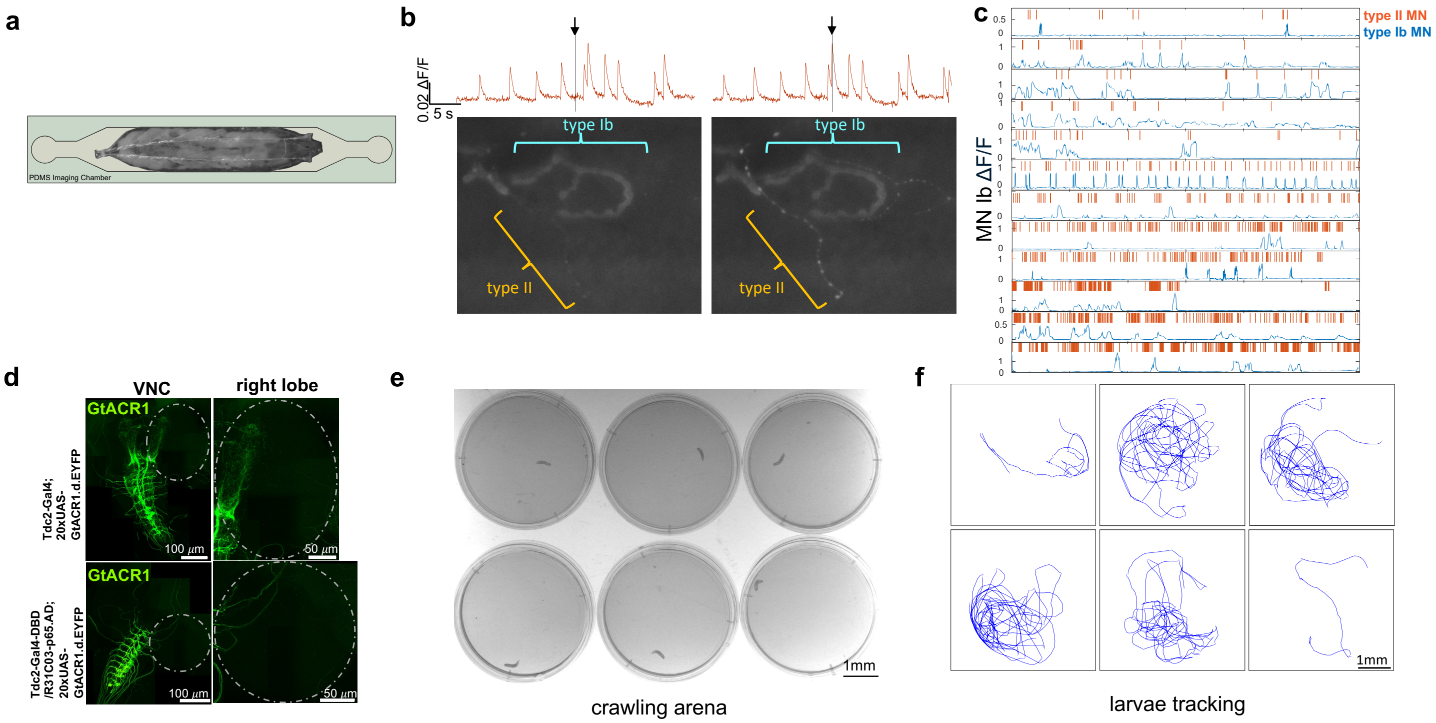

Supplementary Figure 1, related to Figure 1. Tracking of Ib and type II MN activity as well as locomotion.

a) Third-instar larva in a PDMS chamber for simultaneous *in vivo* imaging of activity in type II MNs and synaptic transmission from type I MNs.

b) Cytoplasmic GCaMP6f in type II MNs in dim state (not active, left) and bright state (active, right). Trace above is continuous measure of type II MN change in fluorescence. Arrows show the time point from which each image is taken.

c) Examples from 12 additional animals of *in vivo* imaging (as in **Figure 1b**) showing ticks to indicate starts of type II activity bouts (orange) and their corresponding Ib MN synaptic transmission bouts (blue); animals ordered with higher type II activity lower down.

d) Max intensity projection of pan-OA expression driver TDC2-Gal4 (top) and type II MN specific split-Gal4 driver TDC2-Gal4DBD + #70875p65.AD (bottom) both driving 20xUAS-GtACR1.d.EYFP. Entire central nervous system (VNC and brain) shown on left and zoom-in of the right brain lobe on right. Stained for GtACR1.

e) Locomotion tracking for 6 larvae simultaneously (1 larva per well) in a 6-well crawling arena under IR light.

f) Example larval tracks recorded in crawling arena shown in **(e)**.

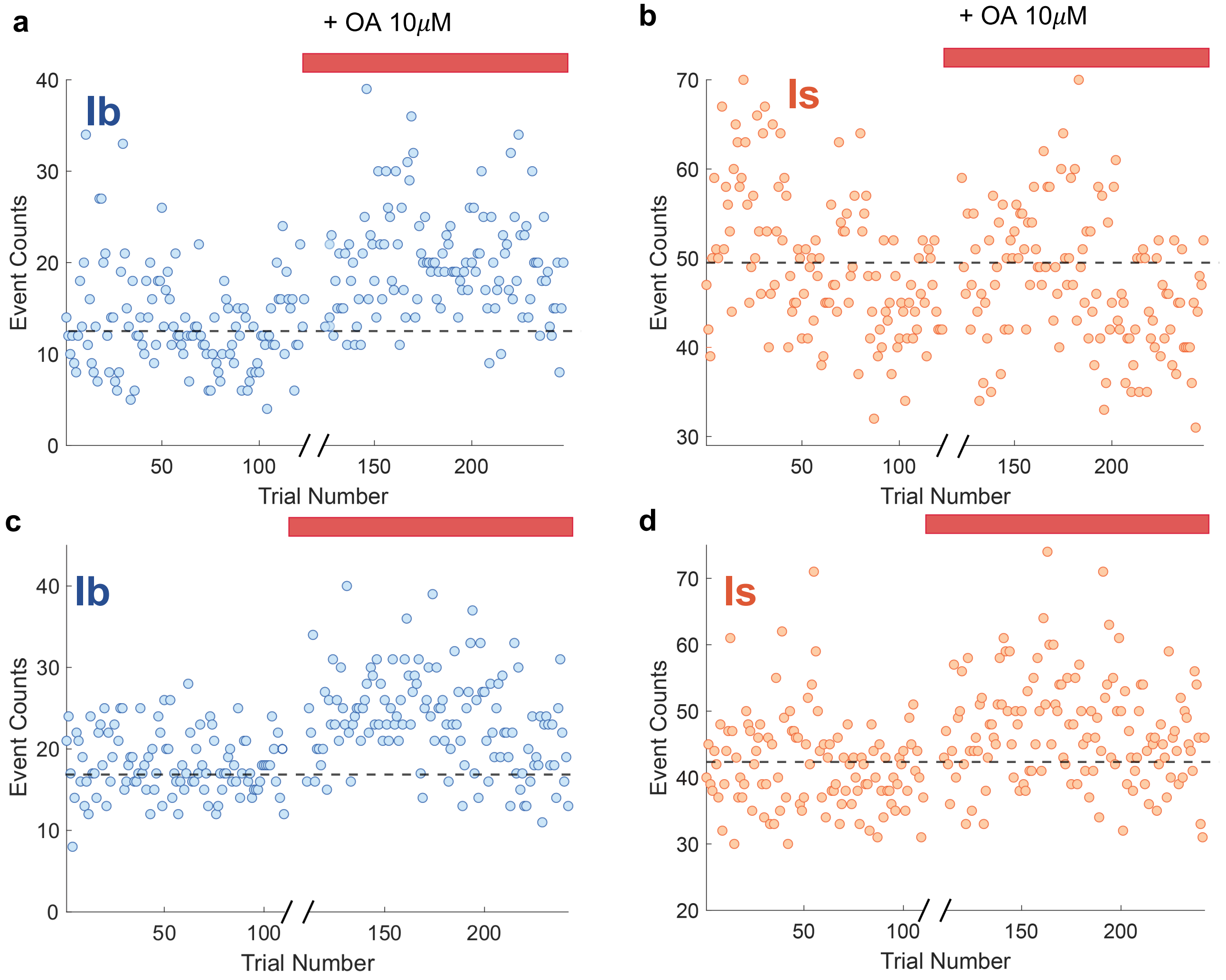

Supplementary Figure 2, related to Figure 2. Effect of octopamine on Ib-Is pairs that innervate muscle 4.
Two additional animals (as in **Fig. 2b**) showing effect of OA on glutamatergic transmission.
a, b) Example animal 1: Event counts per trial for Ib MN **(a)** and Is MN **(b)** converging on same muscle.
c, d) Example animal 2: Event counts per trial for Ib MN **(c)** and Is MN **(d)** converging on same muscle.
Addition of octopamine (OA: 10μM) followed by a 10min incubation period without stimulation during break in x axis and continued during subsequent post-OA stimulation (red bar). Dashed black line indicates mean number of events per trial before OA.

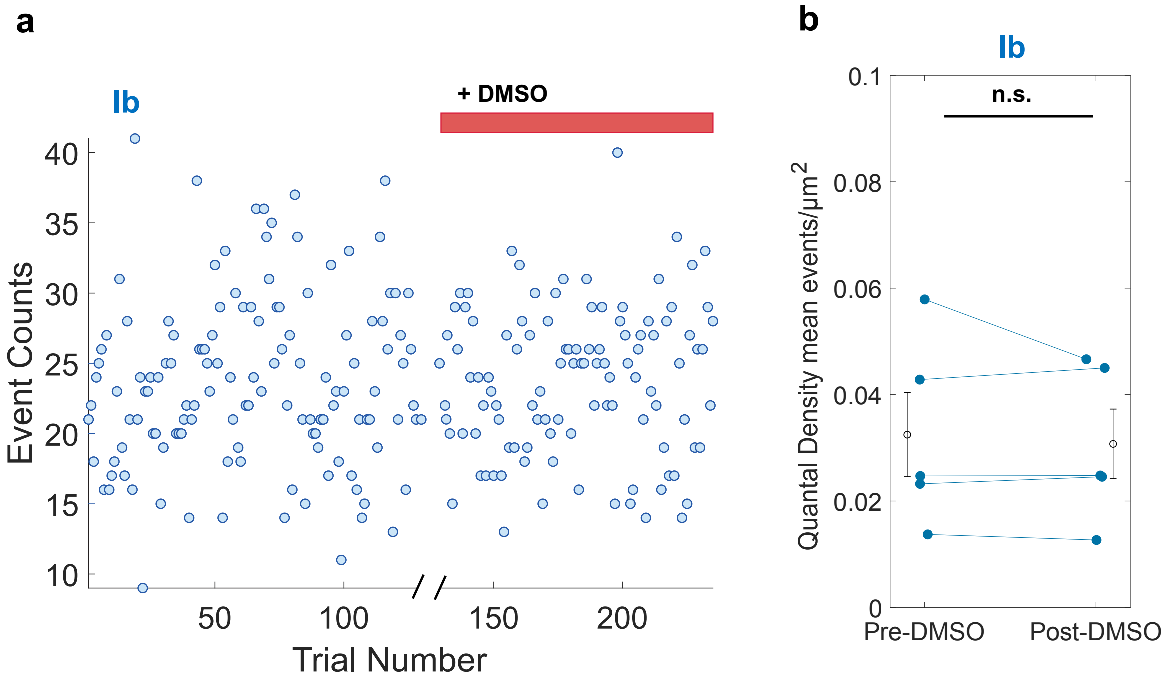

Supplementary Figure 3, related to Figures 2 and 5. DMSO control.

a) Raw ΔF/F glutamate transmission events in response to MN nerve stimulation at 0.2 Hz during 10 min basal period, followed by 6 min interval in 1% DMSO (no imaging, no stimulation), followed by 10 min of transmission imaging during stimulation at 0.2 Hz in continued DMSO.

b) Quantal density (mean events / μm^2) for Ib MNs (n = 5 NMJs). Error bars are mean ± S.E.M. (n.s., not significant by paired t-test).

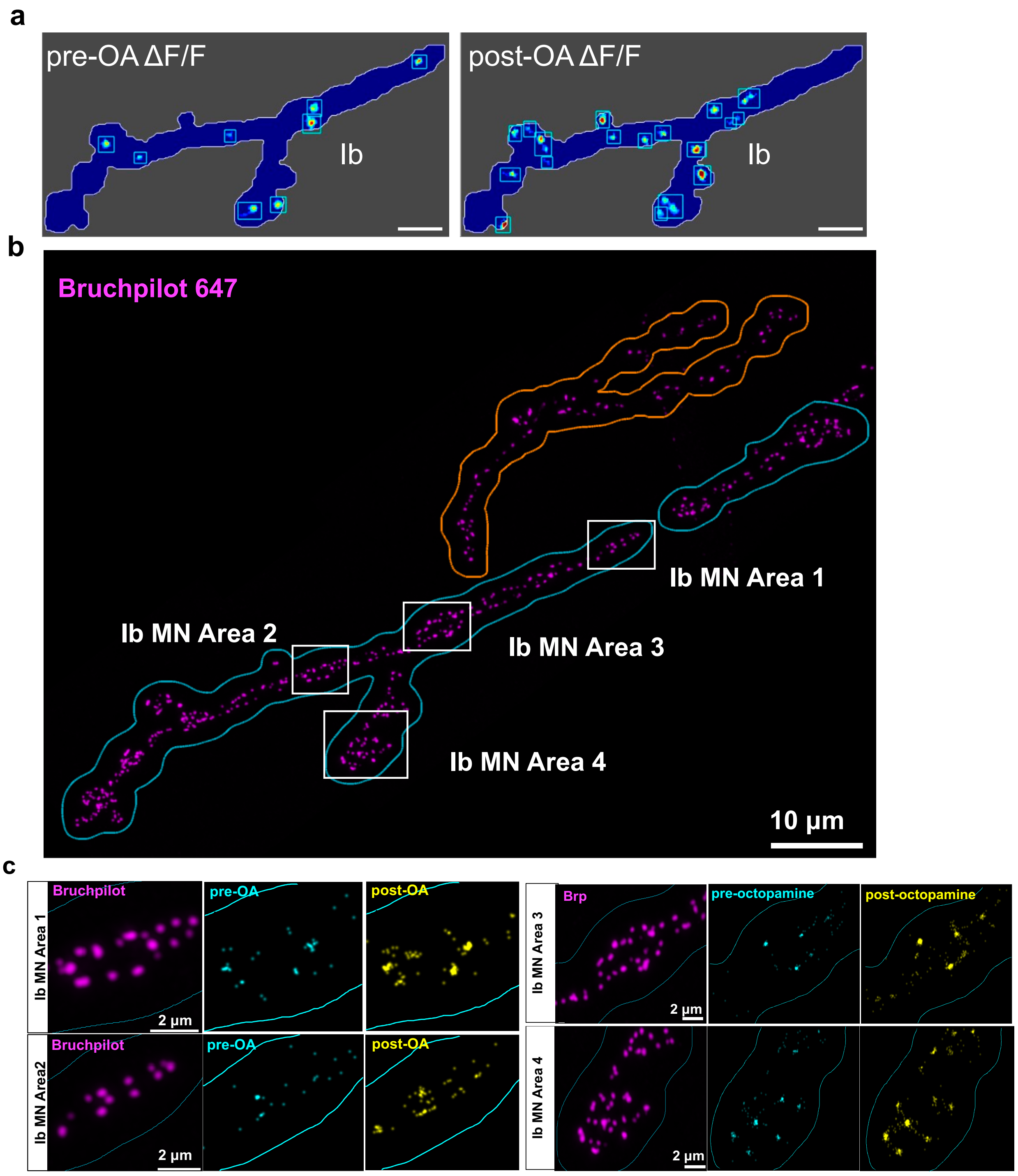

Supplementary Figure 4, related to Figure 2. Effect of octopamine on release from Ib synapses.
a) Left: raw SynapGCaMP6f ΔF/F glutamate transmission events from type Ib MN pre-OA for a single trial. **Right:** raw SynapGCaMP6f ΔF/F glutamate transmission events from the same Ib MN post-OA for a single trial. Boxes show where a ΔF/F transmission events were detected. Scale bar = 10 μm
b) Ib/Is pair stained for the AZ scaffolding protein Bruchpilot (Brp). Blue outline is the Ib MN boundary. Orange outline is the Is MN boundary. Four ROIs indicated for further analysis, below. Scale bar = 10 μm.
c) Four example Ib boutons indicated in (**b**) stained for Brp (left image, magenta) and showing the cumulative positions of centroids of 2D-Guassian fitted ΔF/F glutamate transmission events (see Methods) evoked by 0.2 Hz stimulation before the addition of OA (middle image, transmission events in cyan) and after addition of OA (right image, transmission events in yellow). Scale bars = 2 μm.

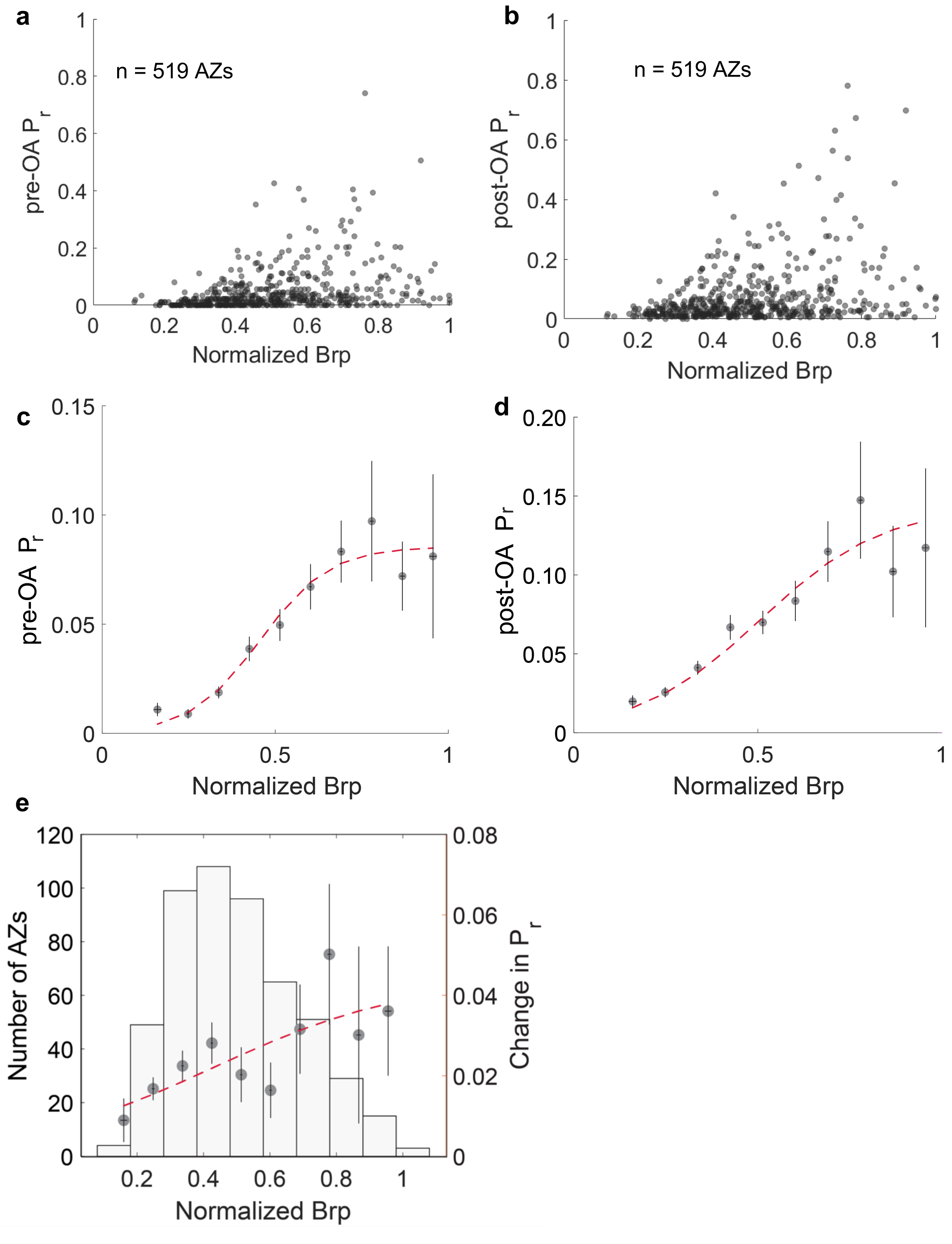

Supplementary Figure 5, related to Figure 2. Relationship of Brp to P_r in type Ib MNs before OA (pre-OA) and after addition of OA (post-OA).

a,b) Pre-OA P_r (a) and post-OA P_r (b) vs. normalized Brp amount for individual AZs from data set in Figure 2c. (n = 519 AZs from 4 NMJs).

c-e) Pre-OA P_r (**c**), post-OA P_r (**d**) and change in P_r (post-OA P_r – pre-OA P_r) (**e**) vs. binned normalized Brp amount (as in **Figure 2h**). Vertical error bars are mean pre-OA P_r ± S.E.M. Horizontal error bars are mean normalized Brp ± S.E.M. Red line is sigmoidal fit, pre-OA P_r: $y = 0.09/(1 + e^{-(x-0.46)*9.9})$. S.S.E. = 4.95 × 10^{-4}; post-OA P_r: $y = 0.13/(1 + e^{-(x-0.44)*6.9})$. S.S.E. = 1.7 × 10^{-3}. Change in P_r: $y = 0.04/(1 + e^{-(x-0.43)*3.4})$. S.S.E. = 5.4 × 10^{-4}.

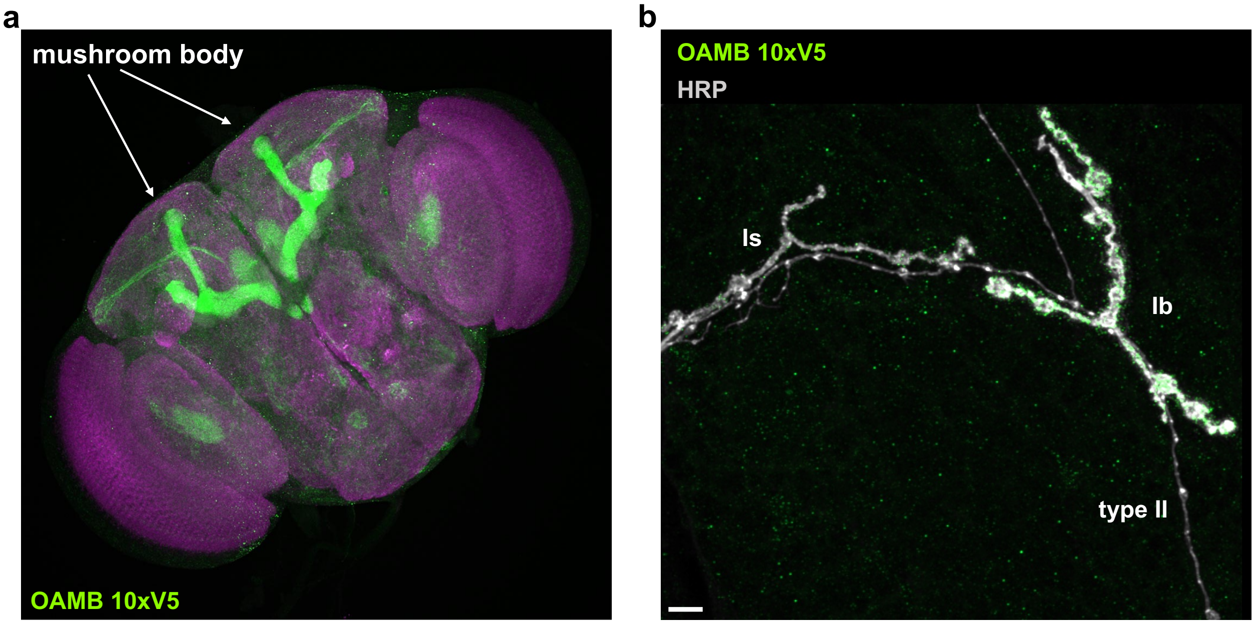

Supplementary Figure 6, related to Figure 3. OAMB receptor staining in the mushroom body and MNs.
a) Mushroom body staining in animals with OAMB receptor CRISPR tagged with 10 copies of the V5 epitope. Staining was against the V5 epitope (green).
b) Same Ib/Is MN pair shown in **Fig**. **3b**, now including anti-HRP staining of axons. The thin long type II MN axon next to the Ib and Is MN has no OAMB expression. Scale bar = 5 μm.

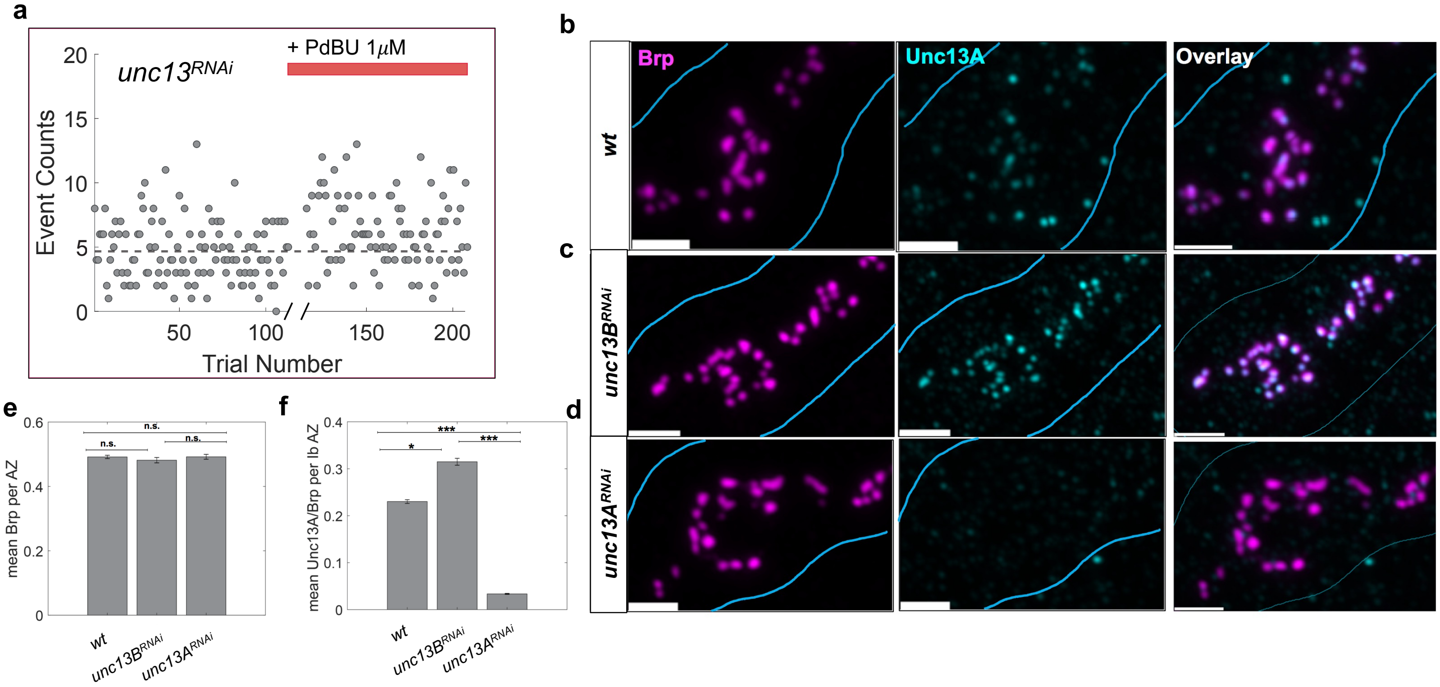

Supplementary Figure 7, related to Figure 6. Combined knockdown of Unc13A and Unc13B eliminated PdBU potentiation and knockdown of Unc13B increases Unc13A protein in type Ib MN active zones.

a) Event counts per trial for Ib MN in an *unc13*^{RNAi} animal in basal period and following addition of PdBU.

b-d) Example boutons from a *wt* Ib MN **(b)**, an *unc13B*^{RNAi} Ib MN **(c)** and an *unc13A*^{RNAi} Ib MN **(d)** stained for Brp **(left)**, Unc13A **(middle)**, with overlay **(right)**. Blue outline is Ib MN boundary. Scale bar = 2 μm.

e,f) Mean normalized Brp per AZ **(e)** and Unc13A/Brp ratio per AZ **(f)** in *wt* and knockdown of Unc13B or Unc13A. Error bars are SEM. (* $p < 0.05$, *** $p < 0.001$, n.s. is not significant by Student's paired t-test, SEM; standard error of the mean).

Supplementary Video 1, related to Figure 1. Tracking of Ib and type II MNs.
Left: Example of live imaging of SynapGCaMP6f in type Ib and cytosolic GCaMP6f in type II MN. **Right:** Activity traces from the video on the left. Type II MN(orange trace, top right) is either in the on or off state. Type Ib MN (blue trace, bottom right) shows the change in the $\Delta F/F$ over time.
